# TMPRSS2 isoform 1 downregulation by G-quadruplex stabilization induces SARS-CoV-2 replication arrest

**DOI:** 10.1186/s12915-023-01805-w

**Published:** 2024-01-08

**Authors:** Alessio De Magis, Philipp Schult, Antonia Schönleber, Rebecca Linke, Kerstin U. Ludwig, Beate M. Kümmerer, Katrin Paeschke

**Affiliations:** 1 Institute of Clinical Chemistry and Clinical Pharmacology, University Hospital Bonn, Venusberg-Campus 1, 53127 Bonn, Germany; 2 Department of Oncology, Haematology and Rheumatology, University Hospital Bonn, Venusberg-Campus 1, 53127 Bonn, Germany; 3 Institute of Human Genetics, University Hospital Bonn, Venusberg-Campus 1, 53127 Bonn, Germany; 4 Institute of Virology, University Hospital Bonn, Venusberg-Campus 1, 53127 Bonn, Germany; 5 German Centre for Infection Research (DZIF), Partner Site Bonn-Cologne, 53127 Bonn, Germany

**Keywords:** SARS-CoV-2, TMPRSS2, Secondary DNA structures, G-quadruplex, Viral replication

## Abstract

**Background:**

SARS-CoV-2 infection depends on the host cell factors angiotensin-converting enzyme 2, ACE2, and the transmembrane serinprotease 2, TMPRSS2. Potential inhibitors of these proteins would be ideal targets against severe acute respiratory syndrome coronavirus type 2 (SARS-CoV-2) infection. Our data opens the possibility that changes within TMPRSS2 can modulate the outcome during a SARS-CoV-2 infection.

**Results:**

We reveal that TMPRSS2 acts not only during viral entry but has also an important role during viral replication. In addition to previous functions for TMPRSS2 during viral entry, we determined by specific downregulation of distinct isoforms that only isoform 1 controls and supports viral replication. G-quadruplex (G4) stabilization by chemical compounds impacts *TMPRSS2* gene expression. Here we extend and in-depth characterize these observations and identify that a specific G4 in the first exon of the *TMPRSS2* isoform 1 is particular targeted by the G4 ligand and affects viral replication. Analysis of potential single nucleotide polymorphisms (SNPs) reveals that a reported SNP at this G4 in isoform 1 destroys the G4 motif and makes TMPRSS2 ineffective towards G4 treatment.

**Conclusion:**

These findings uncover a novel mechanism in which G4 stabilization impacts SARS-CoV-2 replication by changing TMPRSS2 isoform 1 gene expression.

**Supplementary Information:**

The online version contains supplementary material available at 10.1186/s12915-023-01805-w.

## Background

Secondary DNA and RNA structures influence biological processes. Due to their potential in clinical application, the interests towards a specific secondary DNA and RNA structures named G-quadruplexes (G4s) increased in the last years. G4s are non-canonical DNA and RNA secondary structures originated by four repeats of at least two guanines [1, 2]. Since the first demonstration in vitro [1], growing evidence confirmed the formation and the biological function of G4s in vivo [3]. In humans, over 1 million regions were identified that have a strong potential to fold into G4 structures [4–6]. G4 structure-forming sequences (G4 motifs) are enriched at distinct regions such as promoters, transcription factor binding sites, and telomeres [4, 7–9]. Due to their location within the genome, a variety of critical cellular functions like transcription, translation, DSB repair, and telomere maintenance are impacted by G4 formation [10, 11]. The use of G4-specific antibodies (BG4, D1, Sty3) and probes has revolutionized the field and it was possible to detect and visualize G4 structures in vivo by immunofluorescence (IF) microscopy, flow cytometry (FC), and ChIP-seq approaches [7, 12, 13]. Additional molecular, genetic, and different “omic” approaches have also provided convincing evidence for the formation of G4 structures during physiological and pathological processes in living cells, where they are discussed to possess regulatory potential [14–18]. In particular, the observation that multiple oncogenes have a G4 motif in the translated/untranslated regions and, moreover, in their promoter regions [10] opened the possibility that specific stabilization of G4 can be used to target and modulate (positively and negatively) transcription. For multiple oncogenes, e.g., c-MYC [19], KRAS [20], VEGF [21], BCL2 [22], and hTERT [23], it was shown that G4 stabilization suppresses gene expression. In order to chemically induce/stabilize G4 structures, a large variety of DNA/RNA G4 ligands have been developed in the last 20 years [24]. These G4 ligands are currently tested as a novel anti-cancer treatment option [25, 26]. For example, the G4 stabilizer, CX-3564 (Quarfloxin), has completed phase II trials as a candidate therapeutic agent against several tumors, including neuroendocrine tumors, carcinoid tumors, and lymphoma [27]. Also, the G4 ligand CX-5461 is currently at advanced phase II clinical trials for treatment of patients with BRCA1/2-deficient tumors [16].

In addition to eukaryotes, G4 structure-forming sequences have been identified in so far all virus genomes [28]. In these viral genomes, G4 structures have been shown to be important for the viral life cycle [28, 29]. Multiple approaches demonstrated that G4 stabilization by G4 ligands can block viral replication, transcription, and/or translation [28, 29]. These observations led to a current approach to use G4 stabilization as a potential antiviral target against multiple viruses (e.g., hepatitis C virus (HCV), zika virus (ZIKV), and Ebola virus (EBOV)) [28].

At the end of 2019, a new infectious respiratory disease emerged in Wuhan, Hubei province, China [30]. A novel coronavirus, SARS-coronavirus 2 (SARS-CoV-2), closely related to SARS-CoV, was detected in patients and is the etiologic agent of the new lung disease COVID-19 [31]. It has previously been demonstrated that SARS-CoV-2 infection depends on the host cell factors angiotensin-converting enzyme 2, ACE2, and the cellular serine protease, TMPRSS2 [32]. TMPRSS2 is a transmembrane protein that belongs to the serine protease family. For its gene, different spliced variants have been found, each variant encodes different isoforms. Serine proteases are known to be involved in many physiological and pathological processes [33]. TMPRSS2 proteolytically cleaves and activates the viral spike glycoproteins which facilitates virus–cell membrane fusions; spike proteins are synthesized and maintained in precursor intermediate folding states and proteolysis permits the refolding and energy release required to create stable virus–cell linkages and membrane coalescence. In detail, the viral spike (S) proteins are recognized by the SARS-CoV-2 cellular receptor ACE2. After internalization, the serine protease TMPRSS2 induces the S protein priming [32]. Knockdown of TMPRSS2 prevented proteolytic activation and multiplication of influenza A, B viruses [34]. Modulation of *TMPRSS2* expression increased or decreased the sensibility to viral infections [34, 35].

Herein, we identify a novel role of TMPRSS2 during SARS-CoV-2 replication. We determine that a specific G4 structure within the *TMPRSS2* gene impacts the fate of TMPRSS2 isoform expression which has a direct impact on viral replication. We characterize the formation of G4s in *TMPRSS2* using a combination of in silico, in vitro, and *cellulo* assays. Using specific downregulation of TMPRSS2 isoforms, we could characterize the individual functions of the two isoforms. In molecular and biochemical assays, we determine that stabilization of a specific G4 leads to a specific downregulation of isoform 1 which impact viral replication. Known single nucleotide polymorphisms (SNPs) were identified that destroy the G4 motif and prevent G4 formation within *TMPRSS2* which leads to altered TMPRRS2 expression. Together with our results that G4 stabilizing specifically attenuate SARS-CoV-2 replication and that this is coupled to changes within *TMPRRS2* gene expression, we postulate a novel mechanistic model in which G4 stabilization can be used to specifically target viral replication by modulating TMPRSS2 isoform expression.

## Results

### G4s are forming within the TMPRSS2 gene

Multiple experiments have demonstrated a regulatory role of G4 DNA structures during gene expression as well as during protein synthesis. It has been shown that G4s are targeted by proteins and that, depending on the location within the DNA, G4s can either block or stimulate gene expression [10]. Because TMPRSS2 is an essential factor for SARS-CoV-2 entry, we aimed to identify if there are potential G4-forming regions with the genomic region or mRNA of *TMPRSS2*.

**Fig. 1:**
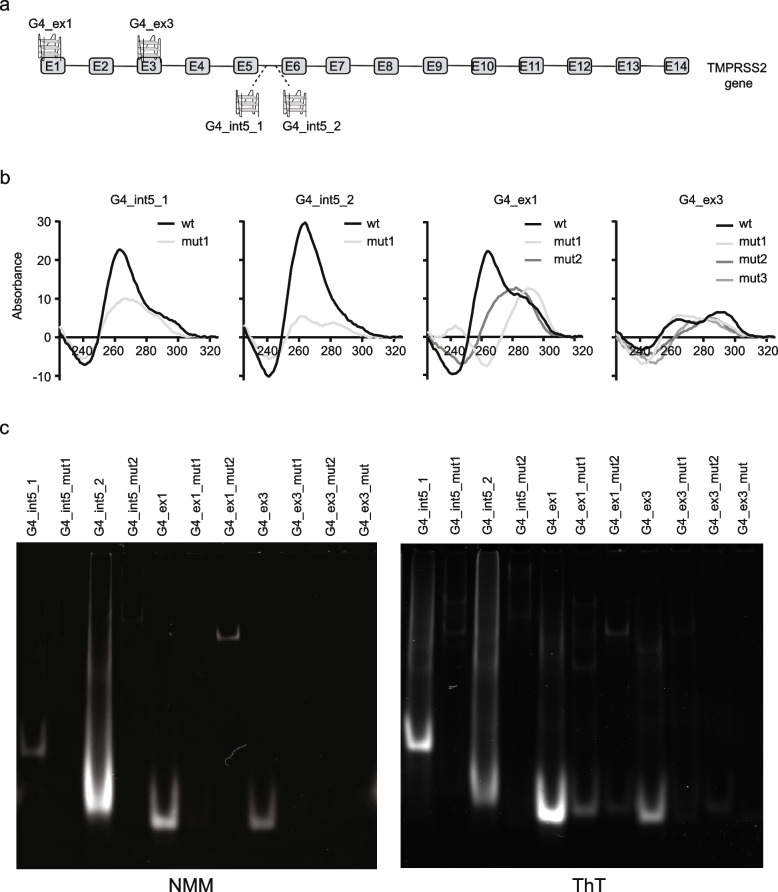
G4s are forming within the TMPRSS2 gene. **a** Schematic illustration of the TMPRSS2 gene and the location of the four G4 motifs identified by G4 Hunter [36]. **b** Circular dichroism of the selected oligonucleotide harboring G4s. In the four panels, the absorbance was calculated in a range between 220 and 330 nm. The black lanes are representative of the original sequence, gray or light gray lanes represent oligonucleotide with mutations in order to disrupt the G4 motifs. **c** NMM (left panel) and ThT (right panel) staining of the selected oligonucleotide harboring G4s, run in a 15% TBE native gels

Using the algorithm G4 hunter [36], we identified 80 putative G4-forming sequences (PQSs) within *TMPRSS2* gene. These PQS have, based on G4 hunter, a G score higher than 2.0, marking those as stable G4 structures (Additional file 1). Based on the presence of a PQS, it is not known whether, why, and when the given PQS form into G4 within TMPRSS2 in living cells. Direct comparison of our PQS with previously experimentally determined G4s [5] identified two PQS that were present in both analysis (Additional file 1). They are located at the fifth intronic region, 29.1 and 37.4 (G4_int5_1 and G4_int5_2, respectively; Additional files 1 and 2). For subsequent analysis, we selected four PQS, the two determined both in silico and in vitro, and two additional ones. The two additional were selected in the coding regions of *TMPRSS2*, based on the highest G score. One at the beginning of the gene (exon 1; G4_ex1) and the second in the exon 3 (G4_ex3; Fig. 1a, Additional files 1 and 2).

Next, we determined in vitro the formation of these four selected PQS. We designed DNA oligonucleotides harboring the G4 motifs as well as control sequences containing mutations within the PQS that are predicted, in silico, to prevent G4 formation. For the intronic regions, several point mutations were introduced to disrupt the G4 motif. For the exonic regions, redundant single point mutations were introduced in order to keep the information of the single codons (Additional file 2). G4 structure formation can be determined by circular dichroism (CD) by specific maxima and minima peaks: parallel G4s have a 264-nm maximum and a 245-nm minimum, antiparallel G4s have a 295 maxima and 260 minima peak [37]. These analyses showed that both intronic G4 structures form a typical parallel G4 structures as identified by the typical maximum peak at 264 nm (Fig. 1b, G4_int5_1; G4_int5_2). The mutated sequences (light gray lanes) did not fold into G4 motifs (Fig. 1b). For the G4 on exon 1 (Fig. 1b, G4_ex1), CD analysis revealed a specific pattern for both parallel (264 nm) and anti-parallel G4 structures (295 nm), suggesting a mixed G4 conformation. A specific point mutation (G4_ex1_mut_1) disrupt the parallel G4 structure. Double point mutations in the second mutant (G4_ex1_mut_2) prevented the formation of both parallel and anti-parallel G4 conformations (Fig. 1b). The G4 on exon 3 (Fig. 1b, G4_ex3) also formed into a mixed G4 structure. A single point mutation in the first mutant (G4_ex3_mut_1) disrupted the anti-parallel G4 structure, but the parallel conformation remained. Double and triple point mutations in the second and third mutant, respectively (G4_ex3_mut_2 and G4_ex3_mut_3), prevented the formation of both parallel and anti-parallel conformations (Fig. 1b). In summary, these analyses confirmed that the predicted G4 motifs can fold into G4 structures and that single point mutations prevent G4 formation.

To strengthen this conclusion, we visualized G4 formation by performing a gel-based assay in vitro. Here, fluorescent dyes, thioflavin T (ThT) and N-methyl mesoporphyrin IX (NMM specific for parallel G4s), that specifically detect G4 structures were used to monitor G4 structure formation [38, 39]. Gel-based assays confirmed the CD analysis that all selected PQS can form into G4 structures in vitro (Fig. 1c). An ethidium bromide staining was used to monitor the amount of DNA loaded in each well (Additional file 1). Similar to CD analysis, mutations of the original sequence prevented the formation of a G4 structures, as indicated by the absence of a distinct band in the ThT gel (Fig. 1c). These results confirmed that all four selected regions can form G4s in vitro and that mutations of the consensus G4 motif eliminates formation of G4s (Fig. 1b,c). Based on these findings, it is likely, due to similarity in the G4 motifs, that among the predicted G4s within *TMPRSS2*, additional will form into G4 structures.

### G4 stabilization by PDS and CX-5461 downregulates expression of TMPRSS2 isoform 1

G4s can influence gene expression by either forming in the promoter or at transcription factor binding sites [10, 14]. G4 ligands have been developed to specifically manipulate gene expression (e.g., oncogenes) [26, 40]. In addition, G4 stabilization by G4 ligands has been extensively tested as a possible anti-viral treatment for different viruses [28]. For subsequent analysis, we have selected two well characterized G4 ligands: CX-5461, currently at advanced phase II clinical trials [16], and PDS which is in depth characterized in vitro and *in cellulo* [9]. Both compounds have been tested in different cell systems and lead to an increase of overall G4 levels in these cells [8, 9, 13, 41]. We characterized the impact G4 formation for TMPRSS2 expression using a breast cancer cell line (MCF-7) and a colorectal cancer cell line (Caco-2), because both cell lines were described to stably express *TMPRSS2* even in unchallenged (i.e., without viral infection) conditions (data: Human Protein Atlas, accessed at http://www.proteinatlas.org). The working concentration of G4 ligands is dependent on the cell type and the length of the treatment. High concentrations of G4 ligands may also cause growth changes and may even induce cell death [8]. To determine the working concentration that induce G4s but is not toxic for the cells, we tested the cytotoxic effects of both selected drugs (PDS and CX-5461) both in Caco-2 and MCF-7. MTT analyses, which assess the metabolic rate of cells as a sign of survival, were performed. We tested different concentrations of PDS (range 1–100 µM) and CX-5461 (range 0.1–20 µM) at different time points, 24, 48, and 72 h (Additional file 3). For both cell lines, longer treatment increased the sensitivity of the drug. However, the reaction towards different treatments varied between the cell lines (Additional file 3). In detail, Caco-2 cells were more sensitive towards CX-5461 while PDS had almost no effect on viability (Additional file 3). Contrary, MCF-7 cells were very sensitive towards PDS, while CX-5461 had almost no effect on viability (Additional file 3). Those results are in line with current models that suggest that different ligands target different G4s and differ in their specificity, binding surface, and cell permeability in a cell type-dependent manner [26]. Taken together, our results suggest a difference tolerance of different cells to G4 stabilization which may indicate that overall different G4 are targeted by the different G4 ligands.

To verify that both PDS and CX-5461 enhance G4 formations in the cells, G4 structures were visualized by IF using the G4 specific antibody, BG4 [12]. IF was performed at different G4 ligand concentrations (CX-5461 (from 0.05 to 2 µM); PDS (from 2 to 50 µM)) and time intervals 24, 48, and 72 h. Note, due to the previously determined high sensitivity of MCF-7 cells towards PDS treatment, in MCF-7 IF, only samples were analyzed after 24 h. Overall, after G4 stabilization, G4 signal increased 1.2- to 1.5-fold for both MCF-7 and Caco-2 cells (Additional files 3 and 4). The most expected results would be a dose-dependent G4-induction in cells. However, the behavior at several time points/concentrations could be explained in two different ways: lower doses of ligands target already all available G4 motifs or G4 ligands lead to changes in proteins that counter balance ligands-induced G4 formations like helicases (e.g., DHX36, BLM,WRN, etc. [42]). Taken together, these data confirmed that in both cell lines, the formation of G4 structures can be modulated by treating cells with either PDS or CX-5461.

G4 stabilization by G4 ligands (e.g., PDS) was demonstrated to impact gene expression of specific genes, e.g., c-MYC [43]. Due to the G4s within *TMPRSS2* gene (Fig. 1), we aimed to address how these two G4 ligands affect the levels of *TMPRSS2*. For this, both cell types were treated with PDS (0–50 µM for 24, 48, and 72 h) or CX-5461 (0.05–2 µM for 24, 48, and 72 h). To monitor gene expression changes specific for *TMPRSS2*, RNA was isolated, transcribed into cDNA, and qPCR analysis was performed using primers specifically targeting *TMPRSS2*.

**Fig. 2:**
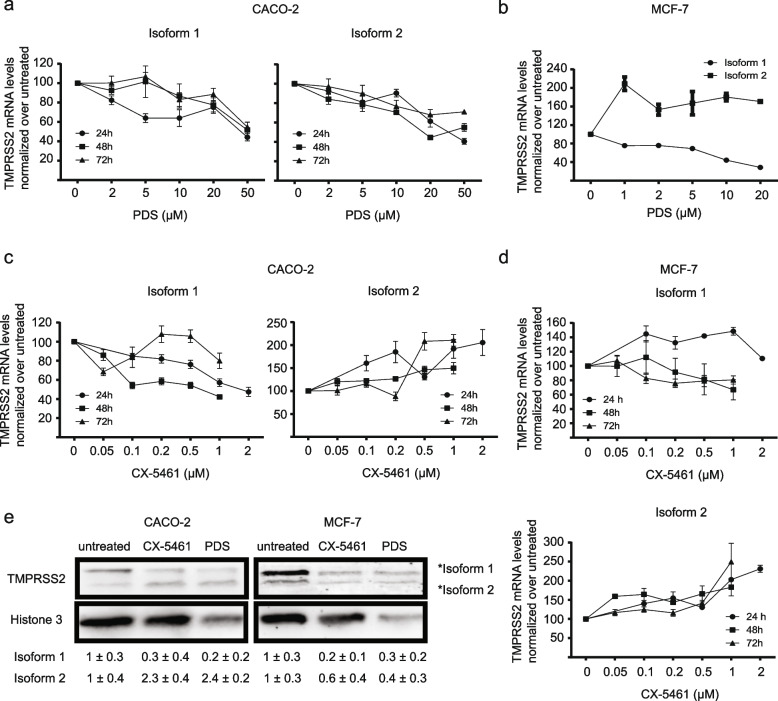
G4 stabilization by PDS and CX-5461 downregulates expression of TMPRSS2 isoform 1. **a** Expression levels of TMPRSS2 isoform 1 (left graph) and isoform 2 (right graph) in Caco-2 cell line treated 24, 48, and 72 h with different concentrations of PDS (0–50 μM). **b** Expression levels of TMPRSS2 isoform 1 and isoform 2 in MCF-7 cell line treated 24 h with different concentrations of PDS (0–20 μM). **c** Expression levels of TMPRSS2 isoform 1 (left graph) and isoform 2 (right graph) in Caco-2 cell line treated 24, 48, and 72 h with different concentrations of CX-5461 (0–2 μM). **d** Expression levels of isoform 1 (upper graph) and isoform 2 (bottom graph) in MCF-7 cell line treated 24, 48, and 72 h with different concentrations of CX-5461 (0–2 μM). mRNA levels in the graphs from **a** to **d** were normalized to the level of U6 snRNA and GAPDH. WT mRNA levels were scaled to 100%. Error bars represent SEM of at least *n* = 3 biological independent experiments. **e** Western blot analysis of protein extracts from Caco-2 (left panel) and MCF-7 (right panel) cells untreated or treated 24 h with 1 μM CX-5461 or 10 μM PDS. Below quantification of *n* = 3 biological independent experiments ± SEM. Membrane was stained with anti-Tmprss2 and anti-Histone 3 antibodies. The original gels are reported in Additional file 9

*TMPRSS2* has two isoforms, both isoforms contain identical transmembrane and extracellular domains but isoform 1 exhibits an extended N-terminal cytoplasmic domain, harboring 37 amino acids (aa) comprising sequence which are not present in isoform 2. We designed specific primer pairs that allowed us to distinguish between expression changes of isoform 1 and 2. Caco-2 cells treated with PDS (0–50 µM for 24, 48, and 72 h) showed a 10 to 60% reduction of both isoforms at, almost, all time points (Fig. 2a), whereas in MCF-7 cells, treatment for 24 h with PDS (0–50 µM) showed a 20 to 60% reduction of isoform 1 and a 50 to 100% increase of isoform 2 after 24 h (Fig. 2b). As described above, due to the previously determined high sensitivity of MCF-7 cells towards PDS, we selected two lower concentrations of PDS in MCF-7 cells to avoid toxic side effects (1–2 µM). In agreement with above findings, also lower PDS doses (1–2 µM) reduced (20 to 50%) *TMPRSS2* levels in MCF-7 cells (Additional file 4). Similar, in Caco-2 cells, treatment with CX-5461 (0.05–2 µM for 24, 48, and 72 h) led to a 10 to 60% reduction of isoform 1 and a 10 to 100% increase of isoform 2 (Fig. 2c). In MCF-7 cells, CX-5461 (0.05–2 µM) led to a 10 to 40% reduction of isoform 1 (opposite trend was obtained at 24 h) and a 10 to 120% increase of isoform 2 (Fig. 2d). The observation that PDS in Caco-2 decreased expression of both isoforms agrees with previous publications in H1299 cells [44] that endogenously expressed ACE2 and TMPRSS2. Contrary, we identified that CX-5461, as well as PDS in MCF-7 cells, decreased only the expression of isoform 1. In summary, we demonstrated that G4 stabilization negatively affects the expression of mainly isoform 1.

It is known that changes on mRNA levels not always correlate with changes in protein levels [45]. Therefore, in the next experiments, we addressed if TMPRSS2 protein levels change also in response to G4 stabilization. Based on viability (Additional file 3), induction of G4 levels (Additional files 3 and 4) and expression changes (Fig. 2a-d), specific time points, and G4 ligand concentrations were selected. We selected for both cell lines 24 h, 10 µM for PDS and 24 h, 1 µM for CX-5461 (Fig. 2e). Western blot analysis using an antibody directed against TMPRSS2 confirmed gene expression analysis. We monitored a specific downregulation of isoform 1 after treatment with either PDS or CX-5461 in Caco-2 cells (Fig. 2e). In MCF-7, treatment with PDS and CX-5461 resulted in a, slight, downregulation of both isoforms (Fig. 2e). In summary, we demonstrated that both TMPRSS2 mRNA expression as well as protein levels are affected due to G4 stabilization. In particular, G4 stabilization affects mRNA and protein level of isoform 1 of TMPRSS2.

### TMPRSS2 isoform 1 downregulation induces SARS-CoV-2 replication arrest

**Fig. 3:**
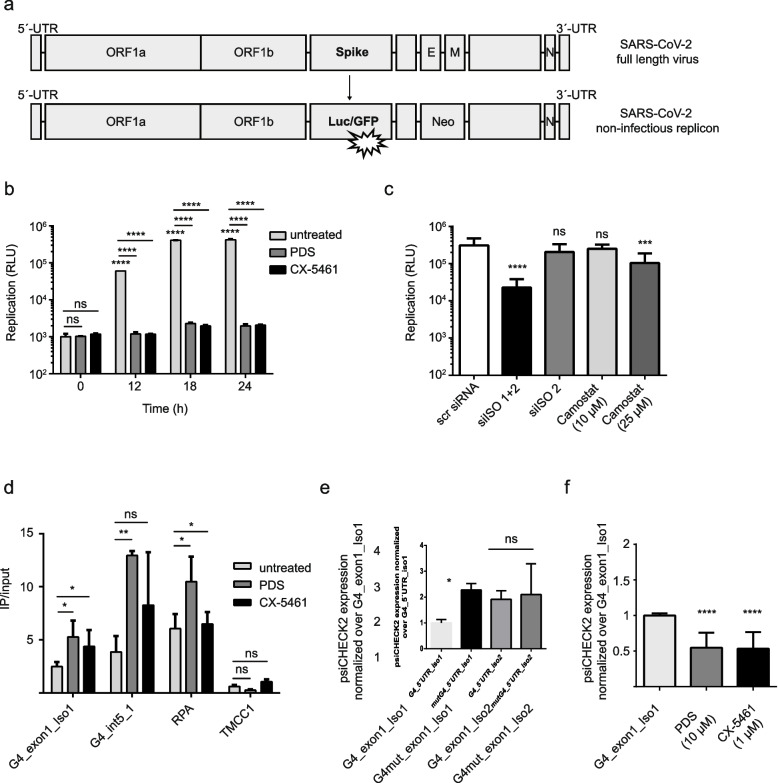
TMPRSS2 isoform 1 downregulation induces SARS-CoV-2 replication arrest. **a** Schematic illustration of the SARS-CoV-2 non-infectious replicon [52] used in the following experiments. **b** Time course experiments of Caco-2 cells pre- treated 24 h with 1 μM CX-5461 or 10 μM PDS and electroporated with the SARS-CoV-2 non-infectious replicon [52]. Luciferase plate was read at time points 0, 12, 18, and 24 h. As a readout were obtained relative lights units (RLU) that reflect the replication of the replicon. **c** SARS-CoV-2 replication in Caco-2 cells transfected with siRNAs against TMPRSS2 isoform 1 and 2, only isoform 2 and scramble siRNA or treated 24 h with 10 and 25 μM camostat. Luciferase activity was measured 24 h after electroporation with the SARS-CoV2 non-infectious replicon [52]. **d** BG4-ChIP-qPCR experiment performed in Caco-2 cells untreated (light gray bars) or treated 24 h with 10 μM PDS (dark gray bars) or 1 μM CX-5461 (black bars). The values in the graph represent the ration between immunoprecipitated chromatin and DNA input. Significance was determined using an ordinary one-way ANOVA multiple comparison. Asterisks indicate statistical significance; in detail, *P < 0.05, **P < 0.01, ***P < 0.001, ****P < 0.0001. **e** Caco-2 cells transfected with psiCHECK™-2 vector containing DNA oligonucleotides harboring the predicted G4 motifs at the exon 1 of the isoform 1, the G-rich sequence in the isoform 2 as well as control mutated sequences cloned upstream of the reporter gene. Expression of psiCHECK™-2 was normalized to the G4_exon1_Iso1 sample. **f** Luciferase assay in Caco-2 cells transfected with psiCHECK™-2 containing the predicted G4 motif at the exon 1 upstream of the reporter gene and either treated for 24 h with 1 μM CX-5461 or 10 μM PDS or left untreated. Expression of psiCHECK™-2 was normalized to the G4_exon1_Iso1 sample

Previous work demonstrated that high levels of TMPRSS2 correlate with strong viral infections because TMPRSS2 supports viral entry into the host cell [32, 35]. Based on our previous observations, we predicted that by targeting G4 structures, we can control the expression of the different isoforms of TMPRSS2 and by this modulate the strength of SARS-CoV-2 infection. Formation of viral G4 structures were shown to impact viral life cycle for example by blocking viral replication [46, 47]. SARS-CoV-2 has multiple G4s within their genome and it was shown that G4 stabilization by ligands can impact SARS-CoV-2 infection [48–51]. However, it is not fully understood if the ligand impacts only viral G4s during SARS-CoV-2 infection or if G4 ligand stabilize and impact multiple G4s from the host cell and the virus. We tested if G4s stabilization by PDS or CX-5461 can modulate SARS-CoV-2 replication. To study viral replication in cells, we used an engineered SARS-CoV-2 replicon, with the capacity of self-replicating without producing infectious virus [52]. This replicon has a luciferase reporter gene instead of the viral spike protein (Fig. 3a), which can be used in luminescence assays. In order to mimic SARS-CoV-2 infection, SARS-CoV-2 specific RNA was in vitro transcribed from the replicon plasmid and electroporated in cells. For subsequent analysis, we focused on Caco-2 cells because in MCF-7 cells, the replicon system was unable to replicate (Additional file 5). Upon electroporation, we monitored viral replication by the change in luciferase expression over time (0, 12, 18, and 24 h). Increased viral replication was detected already 12 h after electroporation, with a maximum between 18 and 24 h (Fig. 3b). Upon G4 stabilization, either by PDS (10 µM) or by CX-5461 (1 µM), a complete replication arrest was documented 12 h after treatment (Fig. 3b).

In Caco-2, as well as in MCF-7 cells, we revealed that G4 stabilization by G4 ligands leads to a robust downregulation of isoform 1 (Fig. 2) and that G4 stabilization impact SARS-CoV-2 replication (Fig. 3b). To test if SARS-CoV-2 replication depends on TMPRSS2 and in particular on one of the isoforms, we downregulated TMPRSS2 by siRNA. For this, we used a pre-designed specific siRNA against both *TMPRSS2* isoforms (si_iso1/2). Further we designed one siRNA against only isoform 2 (si_iso2). Note, due to the high GC content in the 3´-overhang of the isoform 1, it was not possible to design a specific siRNA targeting only isoform 1. Efficient downregulation of TMPRSS2 protein levels were detected 48 h post siRNA transfection by western blot (Additional file 5). Note, after transfection with siRNA against both isoforms, mainly, the isoform 1 levels were decreased, whereas siRNA against only isoform 2 revealed a robust downregulation of only isoform 2 (Additional file 5). Levels were compared to cells transfected with a scramble siRNA (scr siRNA). Next, SARS-CoV-2 replication was monitored after TMPRSS2 downregulation by siRNA by luminescence assay. These analyses revealed a strong decrease in replication after depletion of mainly isoform 1 using si_iso1/2 (Fig. 3c). Downregulation of only isoform 2 alone did not affect viral replication. Same results were obtained in Calu-3 cells, another cell line permissive to SARS-CoV-2 infection (Additional file 5). These results demonstrated that TMPRSS2 activity is not, as previously shown, limited to act during viral entry but also plays a role in the replication of the virus. In particular, this novel function of TMPRSS2 is specific for only the isoform 1 (Fig. 3c). It further drives the hypothesis that G4 stabilization by ligands leads to reduction in viral replication potentially via changing levels of TMPRSS2 isoform 1. Using the full-length virus, knock-down of both isoforms led to a clear decrease in infectious titer. However, also siRNA against isoform 2 alone had a moderate negative effect. This may reflect the necessity of both isoforms for the full viral life cycle (Additional file 10).

It is already known that TMPRRS2 is a viral primase with protease activity [32]. The host cell protease activity of TMPRSS2 can be blocked by the protease inhibitor called camostat mesylate [53]. Upon inhibition of protease activity during SARS-CoV-2 infection, SARS-CoV-2 infection is reduced [53]. We speculated that if TMPRSS2 impacts on viral replication is protease activity dependent, we would also detect a downregulation of viral replication after camostat treatment. To test this speculation, using a SARS-CoV-2 replicon, we monitored viral replication in Caco-2 cells before and after treatment with camostat mesylate (10 and 25 µM). Small changes in viral replication were detected only after 25 µM camostat treatment (Fig. 3c). These experiments confirmed that the protease activity is required for viral entry, whereas viral replication is supported by other functions of TMPRSS2. All together, these results propose a novel role for TMPRSS2 isoform 1 in the SARS-CoV-2 replication, and this activity is independent from the TMPRSS2 protease activity (Fig. 3c). This isoform specificity opens the scenario in which G4 formation can be used to specific target isoform 1.

In order to pinpoint G4 mediated effects on TMPRSS2 isoform expression to a distinct G4, we in-depth re-analyzed G4 motifs in the junction sites of isoform 1 and 2. The difference between isoform 1 and 2 is very small; isoform 1 present a 116 bp in the 3´-overhang and isoform 2 only a 78 bp 3´-overhang. We determined a unique G4 region in the exon 1 of the isoform 1 (future transcribed in the 5´-UTR regulatory region) as well as one G-rich region within the isoform 2 with a low G-score. We designed oligonucleotides harboring the predicted G4 motif in the exon 1 of the isoform 1 and in a G-rich region of isoform 2. Control sequences containing mutations within the G4s that prevent G4 formation were used for all subsequent assays (Additional file 6). By using, as before, ThT in gel staining to confirm G4 structures, we demonstrated that the G4 motif within the exon 1 of the isoform 1 can form a G4 structure in vitro (Additional file 5). Loading was controlled by an ethidium bromide staining (Additional file 5). Mutations of the G4 motif prevented the formation of a G4 structures (Additional file 5). A light band was also detected for G4 within the exon 1 of the isoform 2, but no changes were detected when the G4 motif was disrupted by mutations (Additional file 5). In addition, a second G4 specific probe, NMM, was used in gels, but no parallel G4s were detected in these sequences (Additional file 5). To understand if this G4 is also forming in cells, we performed chromatin immunoprecipitation (ChIP) by targeting G4 structures using the G4 specific antibody BG4. BG4-ChIP followed by qPCRs was performed in Caco-2 cells before and after PDS 10 µM or 1 µM CX-5461 (24 h). As controls, we selected a known G4 positive region (RPA5) and a G4 negative region (TMCC1) as previous published [54]. qPCR analysis showed that the exon 1 of the isoform 1 is significantly enriched and can be co-immunoprecipitated by the G4 antibody (ratio IP/input: 2.5). After G4 stabilization by PDS or CX-5461, this region is even more enriched in the qPCR analysis (CX-5461: ratio IP/input: 4.4, PDS: ratio IP/input: 5.2) (Fig. 3d). Similarly, also a second G4 (studied in Fig. 1: G4_int5_1) located within *TMPRSS2* intron folds in cells as this region can be detected by qPCR analysis using specific primer pairs, before (ratio IP/input: 3.9) and after G4 stabilization by either PDS (ratio IP/input: 13) or CX-5461 (ratio IP/input: 8.3) (Fig. 3d).

In order to characterize the impact of this unique G4 within the exon 1 on *TMPRSS2* isoform 1 transcription or translation, we performed a plasmid-base reporter assay that allows us to monitor transcription and translation changes. For this, we selected a psiCHECK™-2 vector. This system allows us to monitor changes in transcription/translation of a target gene fused to the reporter gene Renilla luciferase. DNA oligonucleotides harboring the predicted G4 motifs of exon 1 isoform 1, the G-rich sequence of isoform 2 as well as the mutated sequences were cloned upstream of the renilla luciferase gene. Caco-2 cells were transfected with these different vector constructs harboring the sequence of interests (SOIs). In Caco-2 cells, we revealed that the G4 of exon 1 decreased expression dramatically, upon mutation of the G4 a 2.5-fold increase in expression was detected (Fig. 3e). As expected, no changes between the original and mutated SOIs were detected after insertion of the G-rich sequence of isoform 2 (Fig. 3e). Same results were obtained in another cell line (HeLa) in the same conditions (Additional file 5). We then examined if PDS or CX-5461 treatment further effects the expression of the reporter construct if the G4 from exon 1 of isoform 1 is used. PDS (10 µM, 24 h) as well as CX-5461 (1 µM, 24 h) treatments led to a twofold decrease in expression (Fig. 3f). All the results suggest that the specific G4 within the exon 1 of isoform 1 has the potential to downregulate gene expression.

### SNPs in the exon 1 (5´UTR) of TMPRSS2 isoform 1 influence protein expression

**Fig. 4:**
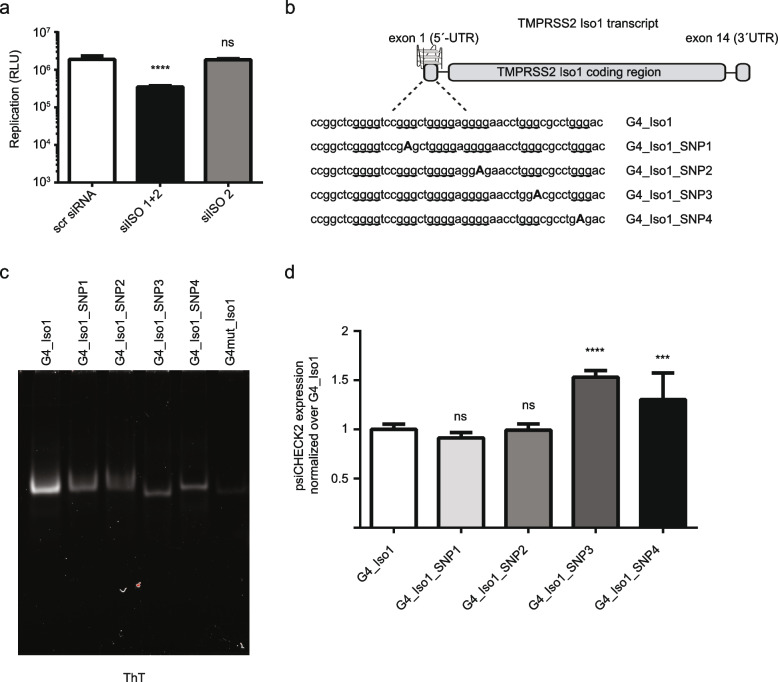
SNPs in the exon 1 (5´UTR) of TMPRSS2 isoform 1 influence protein expression in Caco-2. **a** Yellow fewer virus (YFV) replication in Caco-2 cells transfected with siRNAs against TMPRSS2 isoform 1 and 2 and only isoform 2. Luciferase plate was read 24 h after electroporation with the YFV replicon [56]. **b** Schematic illustration of the G4 at exon 1 of TMPRSS2 gene and the four SNPs that partially disrupt the G4 motifs obtained by NCBI. **c** ThT staining of the oligonucleotide harboring the G4 in the exon1 of the isoform 1 as well as oligonucleotide contained four SNPs that partially disrupt the G4 motifs obtained by NCBI. **d** Caco-2 cells transfected with psiCHECK™-2 containing the G4 at exon 1 of TMPRSS2 gene and the four SNPs that partially disrupt the G4 motifs obtained by NCBI, cloned upstream of the reporter gene. Expression of psiCHECK™-2 was normalized to the G4_ Iso1 sample

In the here presented data, we revealed that isoform 1 of TMPRSS2 impacts SARS-CoV-2 replication (Fig. 3, Additional file 5). To shed light if TMPRSS2 function during viral replication is restricted to members of the family Coronaviridae or if it also modulates replication of other viral families, we have selected a replication system of flaviviruses. Flaviviruses are a large viral family including relevant human pathogens like the dengue virus (DENV), yellow fever virus (YFV), and zika virus (ZIKV) [55]. We used a YFV reporter replicon system to monitor viral replication [56] after downregulation of the specific isoforms of TMPRSS2 (see above) (Fig. 4a). Luminescence assay confirmed a 5.4-fold decrease in replication upon treatment with si_iso1/2 that reduced mainly isoform 1 (Fig. 4a); no changes were determined after downregulation of only isoform 2.

After demonstrating that the isoform 1 of TMPRSS2 not only impacts replication of SARS-CoV-2 but also this of YFV, we raised the hypothesis that changes in viral replication of human individuals after infection might depend on different expression of TMPRSS2 isoforms. We determined if and where SNPs in TMPRSS2 have been observed in the human population and if they are destroying the G4 potential. Using dbSNP (NCBI), we identified four SNPs that are located in the exon 1, all of which are predicted, based on G4 hunter, to reduce the potential of G4 formation: rs1430156730 (SNP1); rs565468881 (SNP2), rs2091471351 (SNP3), and rs2091471316 (SNP4). DNA oligonucleotides harboring the predicted G4 motifs within the exon 1 of the isoform 1 as well as all 4 sequences containing one of the selected SNPs were designed (Fig. 4b; Additional file 7). G4 formation was monitored, by ThT gel analysis, in the control G4 as well as in SNP1-4. These data confirmed that all selected SNPs can partially destabilize the G4 structure in vitro (Fig. 4b,c). DNA loading was controlled by ethidium bromide staining (Additional file 8). These results demonstrated that a single nucleotide change, introduced by a SNP, can impact G4 formation. In order to reveal the direct impact of the selected SNPs on gene expression, we cloned SNP1-4 upstream of the reporter gene in the psiCHECK™-2 vector and checked the expression levels in Caco-2 cells. We predicted, if the selected SNP is relevant for G4 mediated gene expression changes, it will lead to enhanced gene expression of the target region as gene expression is no longer blocked by the G4s. Luminescence assay showed for SNP3 and 4 a 1.5- and a 1.3-fold increase in TMPRSS2 expression, compared to the original G4 sequence of isoform 1 (G4_Iso1) (Fig. 4d). No changes in gene expression were obtained after insertion of SNP1 and SNP2. Similar results were observed in Hela cells; a 2- and a 1.3-fold increase in TMPRSS2 expression were determined for SNP3 and SNP4, respectively (Additional file 8). These results suggest that G4 destabilization, induced by SNPs, can lead to an increase of TMPRSS2 expression. Based on these data, we conclude that selected SNPs in the exon 1 of TMPRSS2 will impact G4 formation.

## Discussion

**Fig. 5:**
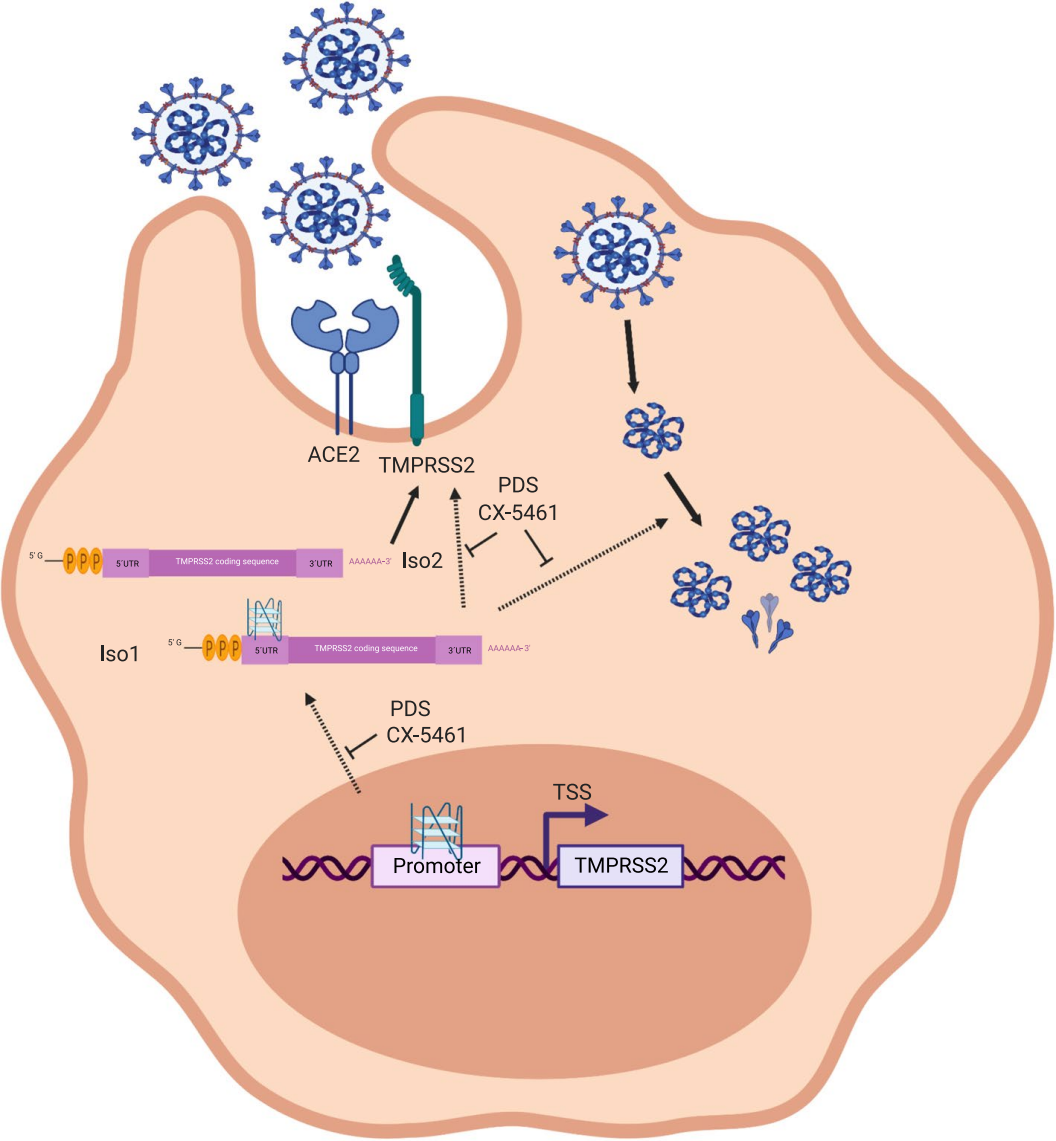
Model. PDS and CX-5461 treatments stabilize G4s in the promoter and gene body inducing a partial downregulation of the TMPRSS2 mRNA, especially of the isoform 1. Furthermore, G4 stabilizers treatment induces stabilization of the G4 in the 5´-UTR (exon 1) of the isoform 1 with a consequent downregulation of the TMPRSS2 isoform 1 protein. TMPRSS2 isoform 1 downregulation will be crucial for inhibiting the replication of the already internalized SARS-CoV-2 replicon

In the last decade, increased attention arose around G4 DNA structures because of their roles in key biological processes, e.g., replication, transcription, and translation [10]. Small molecules able to stabilize DNA/RNA G4s have been developed [24]; these G4 ligands are currently tested as a novel anti-viral treatment option [57]. Here in this work, we aimed to deepen our understanding on TMPRRS2 regulation and how G4 stabilization impacts, via TMPRSS2, SARS-CoV-2 infection. We determined that G4s are forming *in cellulo* and in vitro within *TMPRSS2* gene (Fig. 1, Fig. 3). Stabilization of those G4s by either PDS or CX-5461 directly affected the *TMPRSS2* expression, mainly of isoform 1 (Fig. 2). We conclude that G4 stabilization leads to changes within TMPRSS2 by two pathways, first influencing the transcription by not only modulating promoter activity but also initiating different splicing variant by modulating the expression of, mainly, isoform 1 (Fig. 2, Fig. 5). Our molecular and biochemical experiment identified a new role of TMPRSS2, independent of its known protease activity, during SARS-CoV-2 and YFV viral infection. In particular, we showed that TMPRSS2 supports viral replication (Fig. 3). This new TMPRSS2 function is restricted to isoform 1 (Figs. 3b, 4a) which impacts directly viral replication (Fig. 3b). Those results are in line with previous publications that identified a specific role of the TMPRSS2 isoform 1 in the activation of the influenza A virus hemagglutinin [58]. We identified that the replication inhibition by G4 ligands in Caco-2 cells leads to a stronger repression of viral replication than down-regulation of TMPRSS2 by siRNA (Fig. 3) These results together suggest a complementary activity of the G4 ligands, first in directly blocking viral replication by inducing G4s as a roadblock [10] and by negatively affecting viral replication by reducing the expression of TMPRSS2 isoform 1. In detail, the presence of G4 regions in various viral genomes has been observed: e.g., ZIKV [59], tick-borne encephalitis virus (TBEV) [60], herpes simplex virus 1 (HSV-1) [61], Epstein–Barr virus (EBV) [62], human immunodeficiency virus 1 (HIV-1) [63], Ebola virus (EV) [64], hepatitis C virus (HCV) [65] as well as SARS-CoV-2 [66]). In most of these viruses, G4 stabilization by G4 ligands led to reduced virus production which is connected (dependent on the ligand) to reduced viral replication [67]. We concluded that G4 stabilization modulates viral replication, at least for SARS-CoV-2 and YFV, by a specific reduction of isoform 1 of TMPRSS2. Based on this finding, we speculate that during other virus infections, G4 stabilization has two consequences. First blocking of viral replication by direct G4 formation in the virus genome and second by downregulation of additional host factors that impact viral replication. It has been observed for multiple ligands including PDS and CX-5461 that G4 stabilization block DNA replication both in eukaryotes [46, 47] and viruses, e.g., HCV [68] and ZIKV [69]. Our work extends this model that G4 stabilization leads to a specific downregulation of the host cell factor TMPRSS2, in particular isoform 1, which is important for SARS-CoV-2 replication. It is known that treatment with G4 ligands has a strong impact on gene expression of multiple genes and can also impact splicing events [70] We anticipate that also during other viral infection, G4-induced downregulation of specific host cell factors will impact directly viral infection. This observation highlights the potential of using G4 stabilization to reduce viral replication via two pathways and by this reduce the severeness of viral infection. However, currently, most G4 ligands target multiple G4s, which also induce within the host cell genome instability events and affect gene expression changes and stress responses [8, 41, 71]. We predict that G4 specific ligands for specific G4-forming regions, e.g., the G4 located within the exon 1 of isoform 1, which we characterized, might be an attractive anti-viral tool that will block viral replication but will not impact genome stability events in the host cell.

## Conclusions

Based on our work, we propose that the potential to form G4s within TMPRSS2 can be used as a tool to identify and characterize a risk group that will be more vulnerable for severe SARS-CoV-2 infections. We identified that the two isoforms of TMPRSS2 differ for the 5´-UTR region, where we determined a strong G4 motif that can be targeted by PDS or CX-5461 (Fig. 3e,f). We identified that two putative SNPs in the 5´-UTR of the TMPRSS2 isoform 1 can partially disrupt the G4 motif (Fig. 4c). As a consequence of this SNP, the G4 can no longer form and *TMPRSS2* expression increases significantly (Fig. 4d). SNPs and G4s have been previously correlated genome wide [72, 73] or at specific loci [74].

In summary, our data show that G4 stabilizing by ligands has two effects, first on viral replication and second by modulating the expression of TMPRSS2 isoforms (Fig. 5). To target both viral entry and viral replication, a potential future strategy against viral infection (e.g., SARS-CoV-2) maybe is to treat simultaneously patient with inhibitor of the proteolytic activity (e.g., camostat mesylate) as well as regulators of the TMPRRS2 gene transcription/translation (e.g., G4 stabilizing ligands). Further, the here presented work leads to a future model where the characterization of SNPs that alter the G4-forming potential within genes, e.g., TMPRSS2 may give insights into the risks of patients during SARS-CoV-2 infection, as our data showed that due to changes in G4 formation, potential viral replication is significantly modified.

## Methods

### Circular dichroism

Fifty micrograms of oligonucleotides (Sigma) were diluted in 1 × G4 folding buffer (10 mM Tris–HCl pH 7.5, 0.1 M KCl). The samples were incubated at 95 °C for 5 min and let slowly renatured ON at RT. Absorbance was acquired using a Jasco spectropolarimeter with the following setting:

- Measurement range: 220–330 nm
- Data pitch: 1 nm
- Band width: 2 mm
- Sec response: 0.5
- Standard sensitivity
- Scanning speed: 200 nm/min

### NMM and ThT gel

2.5 μg oligonucleotides (Sigma) were diluted in 1 × G4 folding buffer (10 mM Tris–HCl pH 7.5, 0.1 M KCl). The samples were incubated at 95 °C for 5 min and let slowly renatured ON at RT; 10 μL were mixed with 2.5 μL 5 × native loading dye and the samples loaded on 15% TBE native gel for about 90 min at 80 V. Note, the gel was pre-run for 15 min at 80 V and the wells rinsed thoroughly. The gel was incubated with 10 μg/mL NMM or ThT in 1 × G4 folding buffer for 15 min under agitation and protected from light. The NMM or ThT signal was detected with a ChemiDoc (Biorad). Then the gel was incubated with 0.5 μg/μL ethidium bromide in 1 × G4 folding buffer for 15 min in agitation to stain the total DNA. The ethidium bromide was detected with a ChemiDoc (Biorad).

### Cell lines and cell culture

HeLa cells were purchased from ATCC. Caco-2 and MCF-7 were kindly provided by the Bartok and Feldmann lab (both University Hospital Bonn), respectively. Calu-3 cells were kindly provided by Stefan Pöhlmann (German Primate Center, Göttingen, Germany) through Florian I. Schmidt lab (University of Bonn, Germany). Caco-2, Calu-3, HeLa as well as MCF7 cells were grown in Dulbecco’s modified Eagle’s medium (DMEM) (Gibco™) supplemented with 10% (v/v) fetal bovine serum (FBS, Gibco™) 100 U ml − 1 penicillin–streptomycin (Gibco™). All cell lines were passaged 2–3 times a week and incubated at 37 °C in 5% CO2.

### Cell survival—MTT assay

Cytotoxicity of PDS and CX-5461 was determined with a MTT assay. Seeding was performed in 96-wells plates. After treatment with different concentrations of PDS and CX-5461, cells were washed with PBS and fresh medium containing 500 μg ml-1 of thiazolyl blue tetrazolium bromide solution (Sigma) was added to each well and incubated for 4 h in an incubator at 37 °C in 5% CO2. Medium was subsequently removed, and precipitated formazan crystals were solubilized in 100 μl dimethylsulfoxide (DMSO). Absorbance at 570 nm was measured using a multiplate reader. Cell survival directly correlated with the absorbance values at 570 nm. Absorbance was then normalized against untreated cells (negative control) and used to obtain a compound concentration with a cell viability ≥ 80%.

### BG4 purification

The plasmid expressing an engineered antibody specific to G4 (BG4) [12] was kindly provided by S. Balasubramanian (University of Cambridge, UK). The plasmid was transformed into BL21(DE3) competent cells. BG4 antibody was purified as described in [75]. BG4 antibody was quantified on a NanoDrop spectrophotometer (Thermo Scientific) and stored at − 80 °C. Purity of the BG4 preparation was monitored by SDS-PAGE and ELISA.

### BG4 immunofluorescence

BG4 immunofluorescence was performed as previously described [13]. Briefly, cells were seeded in 6- or 24-multiwell plates; 24 h post seeding cells were treated with PDS and CX-5461 and pre-fixed with a 50/50 solution of DMEM and methanol/acetic acid (3:1) at RT for 5 min. After a brief wash with methanol/acetic acid (3:1), the cells were fixed with methanol/acetic acid (3:1) at RT for 10 min. Cells were then permeabilized with 0.1% (v/v) Triton X-100 in PBS at RT for 3 min under gentle rocking and incubated with blocking solution (2% (w/v) dry milk in PBS, pH 7.4) for 1 h at RT under gentle rocking. Afterwards, cells were incubated in blocking solution containing 0.5/1 μg of BG4 antibody per slide and kept 2 h at RT. Cells were then incubated with blocking solution containing 1:800 rabbit polyclonal antibody against the DYKDDDDK epitope (Cell Signalling ref #2368) for 1 h at RT under gentle rocking. Next, cells were incubated at RT with blocking solution containing 1:1000 fluorescent secondary anti-rabbit IgG (Life technologies ref #A10520) for 1 h at RT under gentle rocking. After each step, cells were washed three times with 0.1% (v/v) Tween-20 in PBS for 10 min. The cover glasses were mounted with a drop of Fluoroshield mounting media solution (Merck) containing the DNA staining fluorophore DAPI.

### BG4 ChIP

BG4 immunoprecipitation was performed as previously described [54].

### Western blot analysis

For standard protein analysis, protein lysates were obtained by lysing the cells in NP-40 lysis buffer or crude 1 × laemmli buffer supplemented with Benzonase. Proteins were running on SDS-PAGEs (8–15%) and blotted on a nitrocellulose membrane (GE Healthcare). After saturating free binding sites with 5% non-fat milk powder in 1X TBS-T, the membrane was incubated with TMPRSS2 (Santa Cruz ref# sc-515727) and Histone 3 (abcam ref# ab1791) antibody overnight at 4 °C under rotation. After three times 10 min washing with 1 × TBS-T, membrane was incubated with matching HRP-coupled secondary antibodies (anti-mouse or anti-rabbit (Santa Cruz Biotechnology) for 1 h at RT followed by another three washing steps. Signals were detected by chemiluminescence of HRP-coupled secondary antibodies (Santa Cruz Biotechnology) on a Gel Doc (Biorad). Uncropped blots are provided in the Source Data file instructions.

### Quantitative PCR

Quantitative PCR (qPCR) was performed using the iQ SYBR Green Supermix (Biorad ref # 1708887).

**Table:** 

TMPRSS2 Transcript 1	Fw GAGTTCAAAGCCATCTTGCTG
	Rv GTGAAAGCGGGTGTGAGG
TMPRSS2 Transcript 2	Fw GGTCCTACTCACCAGGCAGA
	Rv GCTCCCCAAGACACATCCTA

Fold enrichment of the specific transcript of interest was normalized over housekeeping transcript RNU6 and GAPDH used in a previous publication [41]. Microsoft Excel and GraphPad Prism 6.2 were used to plot the graphs.

### siRNA transfection

Twenty-four hours after seeding, HeLa and Caco-2 cells were forward transfected with 100 pM siRNA specific for both isoforms of TMPRSS2 (Thermo ref #SASI_Hs01_00072211) and a custom-made only for the isoform 2 (sense—AGCUAAGCAGGAGGCGGAGGCdTdT) as well as scramble siRNA using Lipofectamine™ RNAiMAX Transfection Reagent (Thermo Fisher ref #13778075). Protein knockdown was assessed by western blot of nuclear proteins extracted from cells 48 h post transfection.

### In vitro* transcription*

pSMART-T7-scv2-replicon was linearized with NotI (NEB) and purified by phenol:chloroform extraction and ethanol precipitation. In vitro transcription and capping were performed using the mMESSAGE mMACHINE T7 transcription kit (Thermo Fisher), according to manufacturer’s instructions; 4 μg of linearized DNA and 15 μL of GTP in a total reaction volume of 100 µl was incubated at 37 °C for 2.5 h. Next, 5 μL of TURBO DNase was added and incubated at 37 °C for 15 min to remove the template. The RNA was then purified by phenol: chloroform extraction and isopropanol precipitation at RT to remove unincorporated nucleotides. The pellet was dissolved in RNAse-free water and quantified by Nanodrop.

### SARS-CoV-2 replicon transfection and luciferase assay

One million cells were electroporated with 2 μg of retrotranscribed RNA originated from the SARS-CoV-2 replicon in the presence of ATP and glutathione in a Gene Pulser Xcell Electroporation System (Biorad) with the preinstalled setting for HeLa cells; 24 h post-electroporetion, cells were resuspended in lysis buffer provided in the luciferase assay kit (Promega). Cells were transferred to 96-well plate and mixed with an equal amount of luciferase reagent. Following incubation of 10 min to achieve full enzymatic activity, firefly luminescence was measured using a plate reader. Samples were measured in technical duplicates.

### Vector cloning

Oligonucleotides were cloned into the psiCHECK™-2 reporter plasmid using the restriction free cloning method. The template was inserted into the vector downstream of the HSV-TK promoter in front of a firefly luciferase gene. The cloning insert was composed of the sequence of interest (SOI) flanked by sequences complementary to the plasmid’s desired cloning site. Using two rounds of polymerase chain reaction (PCR), the insert primer was amplified and extended. The PCR reactions were performed using the Phusion High-Fidelity DNA Polymerase (Thermo Scientific ref # F548L). Finally, the template plasmid was digested by the restriction enzyme DpnI followed by vector transformation into DH5a competent cells. Positive clones were confirmed by sequencing.

### Luciferase assay

Caco-2, HeLa, and MCF-7 cells were seeded into 24-multiwell plate; 24 h post seeding, the psiCHECK™-2 reporter plasmid containing the SOI was transfected using the Lipofectamine 2000 Reagent (Thermo Scientific ref #11668019) according to the manufacturer protocol; 24 h post-transfection, cells were resuspended in lysis buffer provided in the luciferase assay kit (Dual-Glo® Luciferase Assay System Promega ref #E1910). Cells were transferred to 96-well plate and mixed with an equal amount of Dual-Glo Luciferase Reagent (Dual-Glo Luciferase Buffer substituted with Dual-Glo Luciferase Substrate (1:50)). Following incubation of 10 min to achieve full enzymatic activity, firefly luminescence was measured using a plate reader. Afterwards, Dual-Glo Stop & Glo Reagent (Dual-Glo Stop & Glo Buffer substituted with Dual-Glo Stop & Glo Substrate (1:50)) was added to the equal volume of the original sample volume. Following additional 10 min of incubation, Renilla luminescence was measured. The ratio of firefly luminescence to Renilla luminescence was calculated and normalized to the ratio of a control sample. Samples were measured in technical duplicates.

### SARS-CoV-2 infection and plaque assay

CaCo2 cells transfected with siRNA for 48 h were infected with SARS-CoV-2 (Wuhan/6145) at an MOI of 0.01. After 48 h, 10-fold dilutions of the supernatant were used to determine infectious titers by plaque assay in Vero E6 cells overlaid with 1.5% methyl cellulose media. At 3 days post infection, cells were fixed and crystal violet staining was performed to visualize plaques [76].

### Statistical analyses

Significance was calculated using one-sided Student’s *t*-test and ordinary one-way ANOVA multiple comparison tests. Asterisks indicate statistical significance in comparison with wild-type cells: *p < 0.05, **p < 0.01, ***p < 0.001, ****p < 0.0001. Plotted results were based on the average of *N* = 3 biologically independent experiments. 

### Supplementary Information

**Additional file 1. **Putative G4-forming region in TMPRSS2 gene. a) G4 Hunter analysis output. The input was the TMPRSS2 gene (threshold 2.0). b) IGV browser screenshot containing the TMPRSS2 genomic region, the G4 motifs obtained by G4 seq and, in the bottom part, the four G4 region from the G4 Hunter analysis (red arrows). c) Ethidium bromide (EtBr) staining of the selected oligonucleotide harboring G4s run on a 15% TBE native gel.

**Additional file 2. **DNA oligonucleotides harboring the G4 motifs as well as control sequences containing mutations within the PQS that are predicted, *in silico*, to prevent G4 formation.

**Additional file 3. **Treatments with PDS and CX-5461 induce increase in cell mortality and G4s stabilization. a) Vitality assay (MTT) in Caco-2 (left graph) and MCF-7 (right graph) cell lines treated 24, 48 and 72 h with different concentrations of PDS (0 – 100μM). b) Vitality assay (MTT) in Caco-2 (left graph) and MCF-7 (right graph) cell lines treated 24, 48 and 72 h with different concentrations of CX-5461 (0 – 20μM). c) IF staining of Caco-2 cells, treated 24, 48 and 72 h with different concentrations of PDS (0 – 50μM), and stained with BG4 antibody (green), and DAPI (Nucleus border is defined by white borders). Scale bar, 10 µm. Below, quantification of BG4 signal in the nucleus of the cells. d) IF staining of MCF-7 cells, treated 24 h with different concentrations of PDS (0 – 50μM), and stained with BG4 antibody (green), and DAPI (Nucleus border is defined by white borders). Scale bar, 10 µm. Below, quantification of BG4 signal in the nucleus of the cells. The graphs in c) and d) show mean fluorescence intensity (MFI) levels normalized over untreated cells of *n*=3 biological independent experiments. Horizontal line represents the mean value. Significance was determined using an ordinary one-way ANOVA multiple comparison. Asterisks indicate statistical significance; in detail, *P < 0.05, **P < 0.01, ***P < 0.001, ****P < 0.0001.

**Additional file 4. **Treatments with CX-5461 induce G4s stabilization. a) IF staining of Caco-2 cells, treated 24, 48 and 72 h with different concentrations of CX-5461 (0 – 2μM), and stained with BG4 antibody (green), and DAPI (Nucleus border is defined by white borders). Scale bar, 10 µm. Below, quantification of BG4 signal in the nucleus of the cells. b) IF staining of MCF-7 cells, treated 24, 48 and 72 h with different concentrations of CX-5461 (0 – 2μM), and stained with BG4 antibody (green), and DAPI (Nucleus border is defined by white borders). Scale bar, 10 µm. Below, quantification of BG4 signal in the nucleus of the cells. The graphs in a) and b) show mean fluorescence intensity (MFI) levels normalized over untreated cells of *n*=3 biological independent experiments. Horizontal line represents the mean value. Significance was determined using an ordinary one-way ANOVA multiple comparison. Asterisks indicate statistical significance; in detail, *P < 0.05, **P < 0.01, ***P < 0.001, ****P < 0.0001. c) Expression levels of isoform 1 and 2 in MCF-7 cell line treated 48 and 72 h with 1 or 2μM PDS. mRNA levels in the graphs were normalized to the level of U6 snRNA and GAPDH. WT mRNA levels were scaled to 100%. Error bars represent SEM of at least *n*=3 biological independent experiments.

**Additional file 5. **G4s modulate TMPRSS2 expression and SARS-CoV-2 replication. a) Time course experiments of MCF-7 cells pre- treated 24 h with 1μM CX-5461 or 10μM PDS and electroporated with the SARS-CoV-2 non-infectious replicon [52]. Luciferase plate was read at time points 0, 12, 18 and 24 h. b) Western blot analysis of protein extracts from Caco-2 cells transfected with siRNAs against TMPRSS2 isoform 1 and 2 and only isoform 2. Membrane was stained with anti-Tmprss2 and anti-Histone 3 antibodies. The original gel is reported in Additional file 9 c) SARS-CoV-2 replication in Calu-3 cells transfected with siRNAs against TMPRSS2 isoform 1 and 2, only isoform 2 and scramble siRNA or treated 24 h with PDS (10 μM), CX-5461 (1 μM), and Camostat Mesylate (25μM). Luciferase activity was measured 24 h after electroporation with the SARS-CoV2 non-infectious replicon [52]. d) ThT (left panel) and EtBr (right panel) staining of the oligonucleotide harboring G4s in the exon1 of the isoform 1 and 2 as well as oligonucleotide with mutations in order to disrupt the G4 motifs run in a 15% TBE native gels. e) NMM (left panel) and EtBr (right panel) staining of the oligonucleotide harboring G4s in the exon1 of the isoform 1 and 2 as well as oligonucleotide with mutations in order to disrupt the G4 motifs run in a 15% TBE native gels. f) HeLa cells transfected with psiCHECK™-2 vector containing DNA oligonucleotides harboring the predicted G4 motifs at the exon 1 of the isoform 1, the G-rich sequence in the isoform 2 as well as control mutated sequences cloned upstream of the reporter gene. Expression of psiCHECK™-2 was normalized to the G4_exon1_Iso1.

**Additional file 6. **DNA oligonucleotides harboring the G4 motifs in the exon 1 of the isoform 1 and in a G-rich region of isoform 2. as well as control sequences containing mutations within the PQS that are predicted, *in silico*, to prevent G4 formation.

**Additional file 7. **DNA oligonucleotides harboring the predicted G4 motifs within the exon 1 of the isoform 1 as well as all 4 sequences containing one of the selected SNP.

**Additional file 8. **SNPs in the exon 1 (5´UTR) of TMPRSS2 isoform 1 influence protein expression in HeLa cells. a) EtBr staining of the oligonucleotide harboring the G4 in the exon1 of the isoform 1 as well as oligonucleotide contained four SNPs that partially disrupt the G4 motifs obtained by NCBI. b) HeLa cells transfected with psiCHECK™-2 containing the G4 at exon 1 of TMPRSS2 gene and the four SNPs that partially disrupt the G4 motifs obtained by NCBI, cloned upstream of the reporter gene. Expression of psiCHECK™-2 was normalized to the G4_ Iso1.

**Additional file 9. **Original gels. a) original gels from Fig. 2e. b) original gel from Additional file 5.

**Additional file 10. **Infectious virus production and TMPRSS2 expression. Comparison of virus production (plaque-forming units (PFU)/ml) and knock-down efficacy of TMPRSS2 isoforms (iso1 and iso2) upon treatment with siRNA targeting both isoforms (siISO 1+2) or only isoform 2 (siISO 2). All values were normalized to siCtrl.

**Additional file 11.** Individual data values.

**Additional file 12.** Individual data values.

## Data Availability

All data generated or analyzed during this study are included in this published article and its supplementary information files. All data are also available upon request from the corresponding author.

## Abbreviations

ACE2: Angiotensin-converting enzyme 2
TMPRSS2: Transmembrane serinprotease 2
SARS-CoV-2: Severe acute respiratory syndrome coronavirus type 2
G4: G-quadruplex
SNPs: Single nucleotide polymorphisms
PQSs: Putative G4-forming sequences
ThT: Thioflavin T
NMM: N-Methyl Mesoporphyrin IX
CD: Circular dichroism
si_iso1/2: SiRNA against *TMPRSS2* isoforms 1 and 2
si_iso2: SiRNA against only *TMPRSS2* isoform 2
scr siRNA: Scramble siRNA
ChIP: Chromatin immunoprecipitation
SOIs: Sequence of interests
DENV: Dengue virus
YFV: Yellow fever virus
ZIKV: Zika virus
TBEV: Tick-borne encephalitis virus
HSV-1: Herpes simplex virus 1
EBV: Epstein–Barr virus
HIV-1: Human immunodeficiency virus 1
EV: Ebola virus
HCV: Hepatitis C virus

## References

[1] Gellert M, Lipsett MN, Davies DR (1962). Helix formation by guanylic acid. Proc Natl Acad Sci U S A.

[2] Sen D, Gilbert W (1988). Formation of parallel four-stranded complexes by guanine-rich motifs in DNA and its implications for meiosis. Nature.

[3] Spiegel J, Adhikari S, Balasubramanian S (2020). The structure and function of DNA G-quadruplexes. Trends Chem.

[4] Chambers VS, Marsico G, Boutell JM, Di Antonio M, Smith GP, Balasubramanian S (2015). High-throughput sequencing of DNA G-quadruplex structures in the human genome. Nat Biotechnol.

[5] Marsico G, Chambers VS, Sahakyan AB, McCauley P, Boutell JM, Antonio MD, Balasubramanian S (2019). Whole genome experimental maps of DNA G-quadruplexes in multiple species. Nucleic Acids Res.

[6] Bedrat A, Lacroix L, Mergny JL (2016). Re-evaluation of G-quadruplex propensity with G4Hunter. Nucleic Acids Res.

[7] Hänsel-Hertsch R, Beraldi D, Lensing SV, Marsico G, Zyner K, Parry A, Di Antonio M, Pike J, Kimura H, Narita M, Tannahill D, Balasubramanian S (2016). G-quadruplex structures mark human regulatory chromatin. Nat Genet.

[8] De Magis A, Manzo SG, Russo M, Marinello J, Morigi R, Sordet O, Capranico G (2019). DNA damage and genome instability by G-quadruplex ligands are mediated by R loops in human cancer cells. Proc Natl Acad Sci U S A.

[9] Rodriguez R, Miller KM, Forment JV, Bradshaw CR, Nikan M, Britton S, Oelschlaegel T, Xhemalce B, Balasubramanian S, Jackson SP (2012). Small-molecule-induced DNA damage identifies alternative DNA structures in human genes. Nat Chem Biol.

[10] Rhodes D, Lipps HJ (2015). G-quadruplexes and their regulatory roles in biology. Nucleic Acids Res.

[11] Hänsel-Hertsch R, Di Antonio M, Balasubramanian S (2017). DNA G-quadruplexes in the human genome: detection, functions and therapeutic potential. Nat Rev Mol Cell Biol.

[12] Biffi G, Tannahill D, McCafferty J, Balasubramanian S (2013). Quantitative visualization of DNA G-quadruplex structures in human cells. Nat Chem.

[13] De Magis A, Kastl M, Brossart P, Heine A, Paeschke K (2021). BG-flow, a new flow cytometry tool for G-quadruplex quantification in fixed cells. BMC Biol.

[14] Zyner KG, Mulhearn DS, Adhikari S, Martínez Cuesta S, Di Antonio M, Erard N, Hannon GJ, Tannahill D, Balasubramanian S (2019). Genetic interactions of G-quadruplexes in humans. Elife.

[15] Zimmer J, Tacconi EMC, Folio C, Badie S, Porru M, Klare K, Tumiati M, Markkanen E, Halder S, Ryan A, Jackson SP, Ramadan K, Kuznetsov SG, Biroccio A, Sale JE, Tarsounas M (2016). Targeting BRCA1 and BRCA2 Deficiencies with G-quadruplex-interacting compounds. Mol Cell.

[16] Xu H, Di Antonio M, McKinney S, Mathew V, Ho B, O’Neil NJ, Santos ND, Silvester J, Wei V, Garcia J, Kabeer F, Lai D, Soriano P, Banáth J, Chiu DS, Yap D, Le DD, Ye FB, Zhang A, Thu K, Soong J, Lin SC, Tsai AH, Osako T, Algara T, Saunders DN, Wong J, Xian J, Bally MB, Brenton JD, Brown GW, Shah SP, Cescon D, Mak TW, Caldas C, Stirling PC, Hieter P, Balasubramanian S, Aparicio S (2017). CX-5461 is a DNA G-quadruplex stabilizer with selective lethality in BRCA1/2 deficient tumours. Nat Commun.

[17] Halder R, Riou JF, Teulade-Fichou MP, Frickey T, Hartig JS (2012). Bisquinolinium compounds induce quadruplex-specific transcriptome changes in HeLa S3 cell lines. BMC Res Notes.

[18] McLuckie KI, Di Antonio M, Zecchini H, Xian J, Caldas C, Krippendorff BF, Tannahill D, Lowe C, Balasubramanian S (2013). G-quadruplex DNA as a molecular target for induced synthetic lethality in cancer cells. J Am Chem Soc.

[19] Siddiqui-Jain A, Grand CL, Bearss DJ, Hurley LH (2002). Direct evidence for a G-quadruplex in a promoter region and its targeting with a small molecule to repress c-MYC transcription. Proc Natl Acad Sci U S A.

[20] Cogoi S, Xodo LE (2006). G-quadruplex formation within the promoter of the KRAS proto-oncogene and its effect on transcription. Nucleic Acids Res.

[21] Sun D, Liu WJ, Guo K, Rusche JJ, Ebbinghaus S, Gokhale V, Hurley LH (2008). The proximal promoter region of the human vascular endothelial growth factor gene has a G-quadruplex structure that can be targeted by G-quadruplex-interactive agents. Mol Cancer Ther.

[22] Dexheimer TS, Sun D, Hurley LH (2006). Deconvoluting the structural and drug-recognition complexity of the G-quadruplex-forming region upstream of the bcl-2 P1 promoter. J Am Chem Soc.

[23] Palumbo SL, Ebbinghaus SW, Hurley LH (2009). Formation of a unique end-to-end stacked pair of G-quadruplexes in the hTERT core promoter with implications for inhibition of telomerase by G-quadruplex-interactive ligands. J Am Chem Soc.

[24] Wang YH, Yang QF, Lin X, Chen D, Wang ZY, Chen B, Han HY, Chen HD, Cai KC, Li Q, Yang S, Tang YL, Li F (2022). G4LDB 2.2: a database for discovering and studying G-quadruplex and i-Motif ligands. Nucleic Acids Res.

[25] Balasubramanian S, Hurley LH, Neidle S (2011). Targeting G-quadruplexes in gene promoters: a novel anticancer strategy?. Nat Rev Drug Discov.

[26] Kosiol N, Juranek S, Brossart P, Heine A, Paeschke K (2021). G-quadruplexes: a promising target for cancer therapy. Mol Cancer.

[27] Drygin D, Siddiqui-Jain A, O’Brien S, Schwaebe M, Lin A, Bliesath J, Ho CB, Proffitt C, Trent K, Whitten JP, Lim JK, Von Hoff D, Anderes K, Rice WG (2009). Anticancer activity of CX-3543: a direct inhibitor of rRNA biogenesis. Cancer Res.

[28] Ruggiero E, Richter SN (2018). G-quadruplexes and G-quadruplex ligands: targets and tools in antiviral therapy. Nucleic Acids Res.

[29] Abiri A, Lavigne M, Rezaei M, Nikzad S, Zare P, Mergny JL, Rahimi HR (2021). Unlocking G-quadruplexes as antiviral targets. Pharmacol Rev.

[30] Huang C, Wang Y, Li X, Ren L, Zhao J, Hu Y, Zhang L, Fan G, Xu J, Gu X, Cheng Z, Yu T, Xia J, Wei Y, Wu W, Xie X, Yin W, Li H, Liu M, Xiao Y, Gao H, Guo L, Xie J, Wang G, Jiang R, Gao Z, Jin Q, Wang J, Cao B (2020). Clinical features of patients infected with 2019 novel coronavirus in Wuhan China. Lancet.

[31] Zhu N, Zhang D, Wang W, Li X, Yang B, Song J, Zhao X, Huang B, Shi W, Lu R, Niu P, Zhan F, Ma X, Wang D, Xu W, Wu G, Gao GF, Tan W, China Novel Coronavirus Investigating and Research Team (2020). A novel coronavirus from patients with pneumonia in China, 2019. N Engl J Med.

[32] Hoffmann M, Kleine-Weber H, Schroeder S, Krüger N, Herrler T, Erichsen S, Schiergens TS, Herrler G, Wu NH, Nitsche A, Müller MA, Drosten C, Pöhlmann S (2020). SARS-CoV-2 cell entry depends on ACE2 and TMPRSS2 and is blocked by a clinically proven protease inhibitor. Cell.

[33] Lin B, Ferguson C, White JT, Wang S, Vessella R, True LD, Hood L, Nelson PS (1999). Prostate-localized and androgen-regulated expression of the membrane-bound serine protease TMPRSS2. Cancer Res.

[34] Limburg H, Harbig A, Bestle D, Stein DA, Moulton HM, Jaeger J, Janga H, Hardes K, Koepke J, Schulte L, Koczulla AR, Schmeck B, Klenk HD, Böttcher-Friebertshäuser E (2019). TMPRSS2 is the major activating protease of influenza A virus in primary human airway cells and influenza B virus in human type II pneumocytes. J Virol.

[35] Matsuyama S, Nao N, Shirato K, Kawase M, Saito S, Takayama I, Nagata N, Sekizuka T, Katoh H, Kato F, Sakata M, Tahara M, Kutsuna S, Ohmagari N, Kuroda M, Suzuki T, Kageyama T, Takeda M (2020). Enhanced isolation of SARS-CoV-2 by TMPRSS2-expressing cells. Proc Natl Acad Sci U S A.

[36] Brázda V, Kolomazník J, Lýsek J, Bartas M, Fojta M, Šťastný J, Mergny JL (2019). G4Hunter web application: a web server for G-quadruplex prediction. Bioinformatics.

[37] Randazzo A, Spada GP, da Silva MW (2013). Circular dichroism of quadruplex structures. Top Curr Chem.

[38] Xu S, Li Q, Xiang J, Yang Q, Sun H, Guan A, Wang L, Liu Y, Yu L, Shi Y, Chen H, Tang Y (2016). Thioflavin T as an efficient fluorescence sensor for selective recognition of RNA G-quadruplexes. Sci Rep.

[39] Nicoludis JM, Barrett SP, Mergny JL, Yatsunyk LA (2012). Interaction of human telomeric DNA with N-methyl mesoporphyrin IX. Nucleic Acids Res.

[40] Neidle S (2016). Quadruplex nucleic acids as novel therapeutic targets. J Med Chem.

[41] Sauer M, Juranek SA, Marks J, De Magis A, Kazemier HG, Hilbig D, Benhalevy D, Wang X, Hafner M, Paeschke K (2019). DHX36 prevents the accumulation of translationally inactive mRNAs with G4-structures in untranslated regions. Nat Commun.

[42] Sauer M, Paeschke K (2017). G-quadruplex unwinding helicases and their function in vivo. Biochem Soc Trans.

[43] Yuan JH, Tu JL, Liu GC, Chen XC, Huang ZS, Chen SB, Tan JH (2022). Visualization of ligand-induced c-MYC duplex-quadruplex transition and direct exploration of the altered c-MYC DNA-protein interactions in cells. Nucleic Acids Res.

[44] Liu G, Du W, Sang X, Tong Q, Wang Y, Chen G, Yuan Y, Jiang L, Cheng W, Liu D, Tian Y, Fu X (2022). RNA G-quadruplex in TMPRSS2 reduces SARS-CoV-2 infection. Nat Commun.

[45] Wegler C, Ölander M, Wiśniewski JR, Lundquist P, Zettl K, Åsberg A, Hjelmesæth J, Andersson TB, Artursson P (2019). Global variability analysis of mRNA and protein concentrations across and within human tissues. NAR Genom Bioinform.

[46] Zou M, Li JY, Zhang MJ, Li JH, Huang JT, You PD, Liu SW, Zhou CQ (2021). G-quadruplex binder pyridostatin as an effective multi-target ZIKV inhibitor. Int J Biol Macromol.

[47] Westdorp KN, Terhune SS (2018). Impact of RNA polymerase I inhibitor CX-5461 on viral kinase-dependent and -independent cytomegalovirus replication. Antiviral Res.

[48] Panera N, Tozzi AE, Alisi A (2020). The G-quadruplex/helicase world as a potential antiviral approach against COVID-19. Drugs.

[49] Ji D, Juhas M, Tsang CM, Kwok CK, Li Y, Zhang Y (2021). Discovery of G-quadruplex-forming sequences in SARS-CoV-2. Brief Bioinform.

[50] Cui H, Zhang L (2020). G-quadruplexes are present in human coronaviruses including SARS-CoV-2. Front Microbiol.

[51] Qin G, Zhao C, Liu Y, Zhang C, Yang G, Yang J, Wang Z, Wang C, Tu C, Guo Z, Ren J, Qu X (2022). RNA G-quadruplex formed in SARS-CoV-2 used for COVID-19 treatment in animal models. Cell Discov.

[52] He X, Quan S, Xu M, Rodriguez S, Goh SL, Wei J, Fridman A, Koeplinger KA, Carroll SS, Grobler JA, Espeseth AS, Olsen DB, Hazuda DJ, Wang D (2021). Generation of SARS-CoV-2 reporter replicon for high-throughput antiviral screening and testing. Proc Natl Acad Sci U S A.

[53] Hoffmann M, Hofmann-Winkler H, Smith JC, Krüger N, Arora P, Sørensen LK, Søgaard OS, Hasselstrøm JB, Winkler M, Hempel T, Raich L, Olsson S, Danov O, Jonigk D, Yamazoe T, Yamatsuta K, Mizuno H, Ludwig S, Noé F, Kjolby M, Braun A, Sheltzer JM, Pöhlmann S (2021). Camostat mesylate inhibits SARS-CoV-2 activation by TMPRSS2-related proteases and its metabolite GBPA exerts antiviral activity. EBioMedicine.

[54] Hänsel-Hertsch R, Spiegel J, Marsico G, Tannahill D, Balasubramanian S (2018). Genome-wide mapping of endogenous G-quadruplex DNA structures by chromatin immunoprecipitation and high-throughput sequencing. Nat Protoc.

[55] Pierson TC, Diamond MS (2020). The continued threat of emerging flaviviruses. Nat Microbiol.

[56] Jones CT, Patkar CG, Kuhn RJ (2005). Construction and applications of yellow fever virus replicons. Virology.

[57] Ruggiero E, Richter SN (2023). Targeting G-quadruplexes to achieve antiviral activity. Bioorg Med Chem Let.

[58] Zmora P, Moldenhauer AS, Hofmann-Winkler H, Pöhlmann S (2015). TMPRSS2 isoform 1 activates respiratory viruses and is expressed in viral target cells. PLoS One.

[59] Fleming AM, Ding Y, Alenko A, Burrows CJ (2016). Zika virus genomic RNA possesses conserved G-quadruplexes characteristic of the flaviviridae family. ACS Infect Dis.

[60] Holoubek J, Bednářová K, Haviernik J, Huvarová I, Dvořáková Z, Černý J, Outlá M, Salát J, Konkol’ová E, Boura E, Růžek D, Vorlíčková M, Eyer L, Renčiuk D (2022). Guanine quadruplexes in the RNA genome of the tick-borne encephalitis virus: their role as a new antiviral target and in virus biology. Nucleic Acids Res.

[61] Artusi S, Nadai M, Perrone R, Biasolo MA, Palù G, Flamand L, Calistri A, Richter SN (2015). The Herpes Simplex Virus-1 genome contains multiple clusters of repeated G-quadruplex: implications for the antiviral activity of a G-quadruplex ligand. Antiviral Res.

[62] Murat P, Zhong J, Lekieffre L, Cowieson NP, Clancy JL, Preiss T, Balasubramanian S, Khanna R, Tellam J (2014). G-quadruplexes regulate Epstein-Barr virus-encoded nuclear antigen 1 mRNA translation. Nat Chem Biol.

[63] Perrone R, Butovskaya E, Daelemans D, Palù G, Pannecouque C, Richter SN (2014). Anti-HIV-1 activity of the G-quadruplex ligand BRACO-19. J Antimicrob Chemother.

[64] Wang SR, Zhang QY, Wang JQ, Ge XY, Song YY, Wang YF, Li XD, Fu BS, Xu GH, Shu B, Gong P, Zhang B, Tian T, Zhou X (2016). Chemical targeting of a G-quadruplex RNA in the Ebola virus L gene. Cell Chem Biol.

[65] Wang SR, Min YQ, Wang JQ, Liu CX, Fu BS, Wu F, Wu LY, Qiao ZX, Song YY, Xu GH, Wu ZG, Huang G, Peng NF, Huang R, Mao WX, Peng S, Chen YQ, Zhu Y, Tian T, Zhang XL, Zhou X (2016). A highly conserved G-rich consensus sequence in hepatitis C virus core gene represents a new anti-hepatitis C target. Sci Adv.

[66] Belmonte-Reche E, Serrano-Chacón I, Gonzalez C, Gallo J, Bañobre-López M (2021). Potential G-quadruplexes and i-Motifs in the SARS-CoV-2. PLoS One.

[67] Ruggiero E, Zanin I, Terreri M, Richter SN (2021). G-quadruplex targeting in the fight against viruses: an update. Int J Mol Sci.

[68] Jaubert C, Bedrat A, Bartolucci L, Di Primo C, Ventura M, Mergny JL, Amrane S, Andreola ML (2019). Author Correction: RNA synthesis is modulated by G-quadruplex formation in hepatitis C virus negative RNA strand. Sci Rep.

[69] Majee P, Pattnaik A, Sahoo BR, Shankar U, Pattnaik AK, Kumar A, Nayak D (2021). Inhibition of Zika virus replication by G-quadruplex-binding ligands. Mol Ther Nucleic Acids.

[70] Georgakopoulos-Soares I, Parada GE, Wong HY, Medhi R, Furlan G, Munita R, Miska EA, Kwok CK, Hemberg M (2022). Alternative splicing modulation by G-quadruplexes. Nat Commun.

[71] Miglietta G, Russo M, Duardo RC, Capranico G (2021). G-quadruplex binders as cytostatic modulators of innate immune genes in cancer cells. Nucleic Acids Res.

[72] Baral A, Kumar P, Halder R, Mani P, Yadav VK, Singh A, Das SK, Chowdhury S (2012). Quadruplex-single nucleotide polymorphisms (Quad-SNP) influence gene expression difference among individuals. Nucleic Acids Res.

[73] Gong JY, Wen CJ, Tang ML, Duan RF, Chen JN, Zhang JY, Zheng KW, He YD, Hao YH, Yu Q, Ren SP, Tan Z (2021). G-quadruplex structural variations in human genome associated with single-nucleotide variations and their impact on gene activity. Proc Natl Acad Sci U S A.

[74] Beaudoin JD, Perreault JP (2010). 5’-UTR G-quadruplex structures acting as translational repressors. Nucleic Acids Res.

[75] De Magis A, Götz S, Hajikazemi M, Fekete-Szücs E, Caterino M, Juranek S, Paeschke K (2020). Zuo1 supports G4 structure formation and directs repair toward nucleotide excision repair. Nat Commun.

[76] Koenig PA, Das H, Liu H, Kümmerer BM, Gohr FN, Jenster LM, Schiffelers LDJ, Tesfamariam YM, Uchima M, Wuerth JD, Gatterdam K, Ruetalo N, Christensen MH, Fandrey CI, Normann S, Tödtmann JMP, Pritzl S, Hanke L, Boos J, Yuan M, Zhu X, Schmid-Burgk JL, Kato H, Schindler M, Wilson IA, Geyer M, Ludwig KU, Hällberg BM, Wu NC, Schmidt FI (2021). Structure-guided multivalent nanobodies block SARS-CoV-2 infection and suppress mutational escape. Science.

