# Supplementary material for: TMPRSS2 isoform 1 downregulation by G-quadruplex stabilization induces SARS-CoV-2 replication arrest
Source: BMC Biol. 2024 Jan 8;22:5. doi: 10.1186/s12915-023-01805-w (PMC10773119; doi:10.1186/s12915-023-01805-w)

a

Analysis settings	Analysis results	Sequence info
Window size: 25	Quadruplexes found: 80	TMPRSS2
Threshold: 2	Frequency: 1.8 / 1000 bp	43,631 bp
		GC: 21961 (50.4%)

b

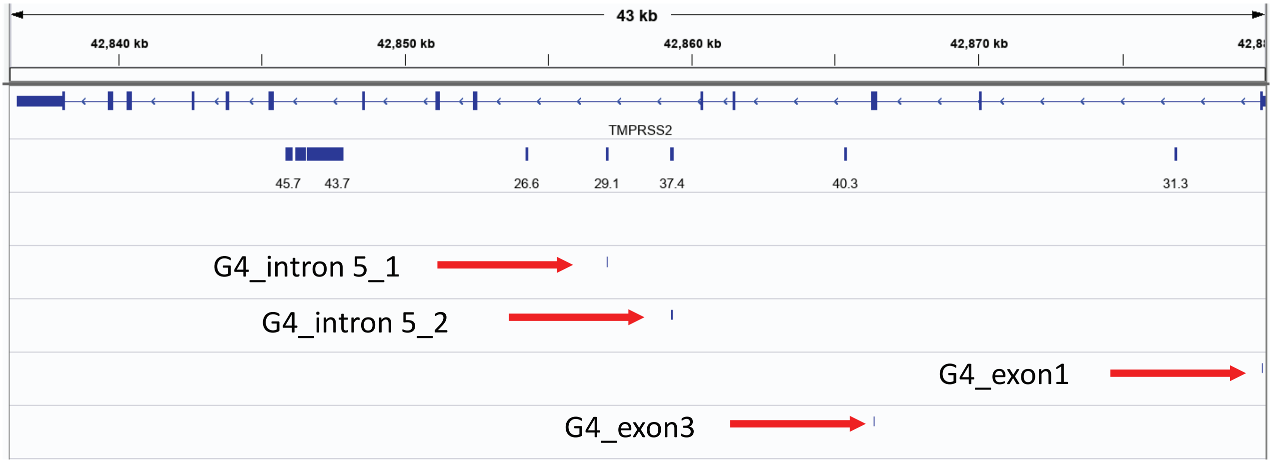

c

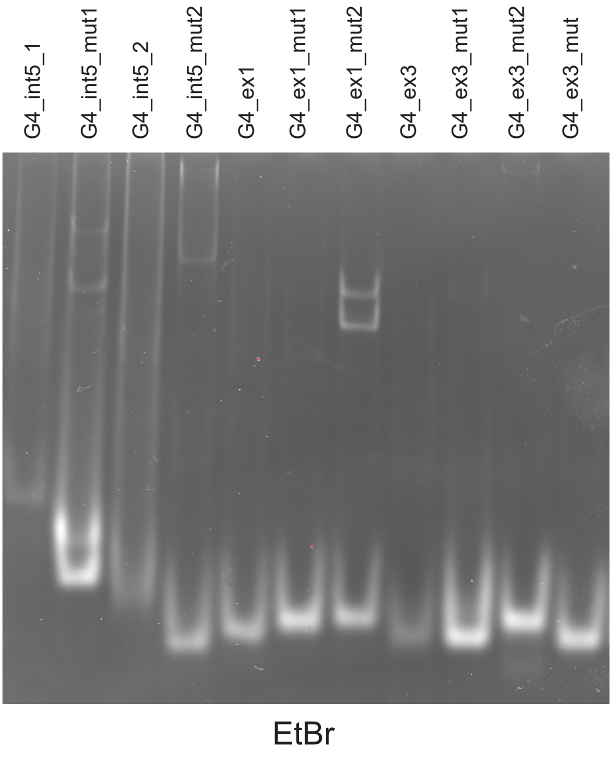

Supplement: Supplementary file 1 — Additional file 1. Putative G4-forming region in TMPRSS2 gene. a) G4 Hunter analysis output. The input was the TMPRSS2 gene (threshold 2.0). b) IGV browser screenshot containing the TMPRSS2 genomic region, the G4 motifs obtained by G4 seq and, in the bottom part, the four G4 region from the G4 Hunter analysis (red arrows). c) Ethidium bromide (EtBr) staining of the selected oligonucleotide harboring G4s run on a 15% TBE native gel. [file 12915_2023_1805_MOESM1_ESM.pdf]
