# Supplementary material for: TMPRSS2 isoform 1 downregulation by G-quadruplex stabilization induces SARS-CoV-2 replication arrest
Source: BMC Biol. 2024 Jan 8;22:5. doi: 10.1186/s12915-023-01805-w (PMC10773119; doi:10.1186/s12915-023-01805-w)
Supplement: Supplementary file 2 — Additional file 2. DNA oligonucleotides harboring the G4 motifs as well as control sequences containing mutations within the PQS that are predicted, in silico, to prevent G4 formation. [file 12915_2023_1805_MOESM2_ESM.pdf]

	sequence
G4_int5_1	TCTGGGGTGTATGGGGGGATGCGGGGTAGG
mut_int5_1	TCTGGGGTGTATGGGGGGATGCCCCGTACG
G4_int5_2	GGGGGCGGGGGGTGACAACACTGGGG
mut_int5_2	GGGGGCGCGAAGTGACAACACTCAGG
G4_ex1	GGGCGGGGCAGGGGGCATCGGCGGGT
mut_G4_ex1_1	GGGCGCGGCAGGGGGCATCGGCCGGT
mut_G4_ex1_2	GCGCGCCGCAGGGGGCATCAGCCGGT
G4_ex3	GGGGCGTACTGGGGCACGGGGGACGG
mut_G4_ex3_1	GGGGCGTACTGCGGCACGGGGGACGG
mut_G4_ex3_2	GGGGCGTACTGCGGCACGGGCGACGG
mut_G4_ex3_3	GGCGCGTACTGCGGCACGGGCGACGG

position	G4 Hunter score	Marsico et al
chr21: 41,778,914 - 41,778,943	2,00	29,1
chr21: 41,781,179 - 41,781,204	2,20	37,4
chr21: 41,801,784 - 41,801,809	2,00	
chr21: 41,801,784 - 41,801,809	2,07	
