# Supplementary figures and images for: TMPRSS2 isoform 1 downregulation by G-quadruplex stabilization induces SARS-CoV-2 replication arrest
Source: BMC Biol. 2024 Jan 8;22:5. doi: 10.1186/s12915-023-01805-w (PMC10773119; doi:10.1186/s12915-023-01805-w)

**a**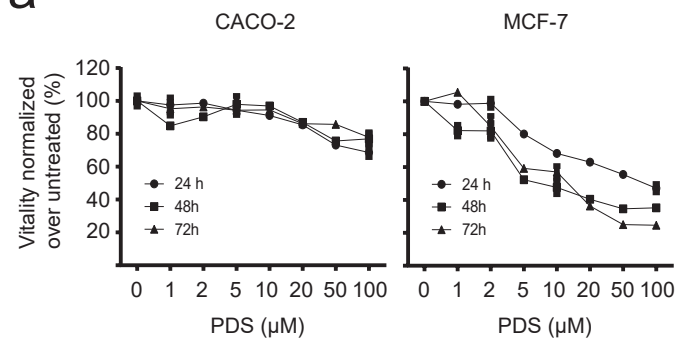**b**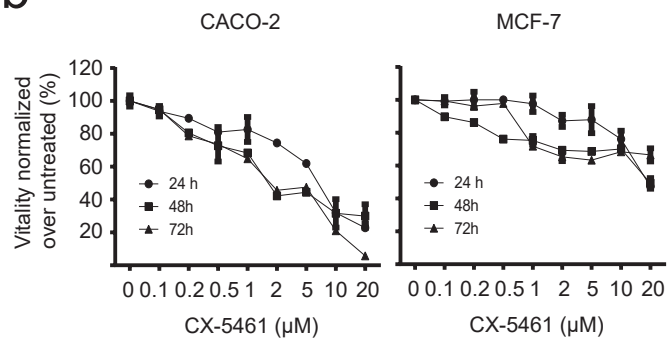**c**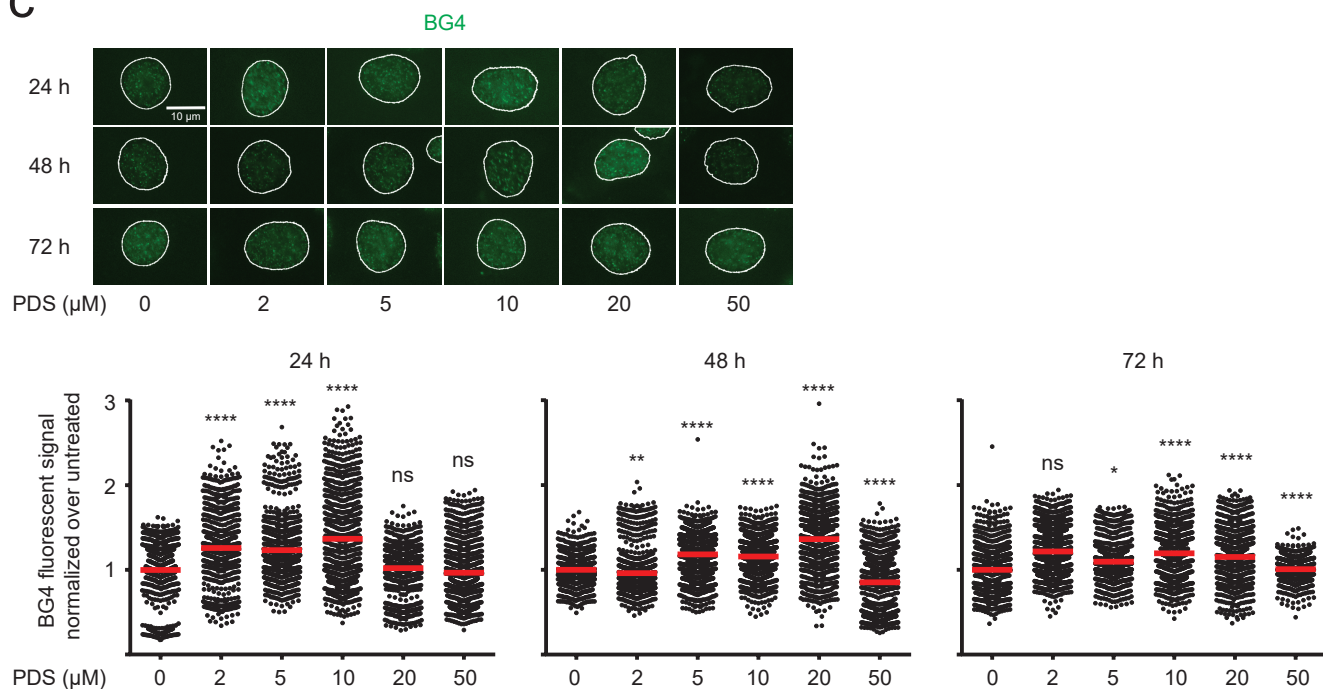**d**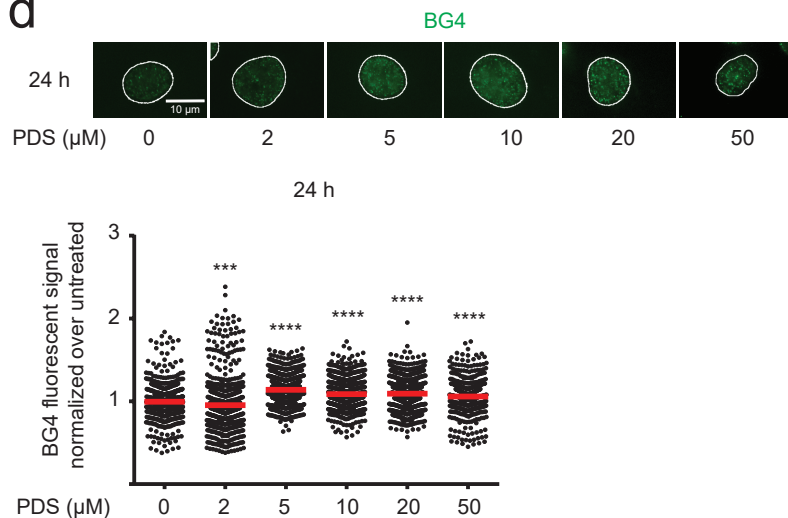

Supplement: Supplementary file 3 — Additional file 3. Treatments with PDS and CX-5461 induce increase in cell mortality and G4s stabilization. a) Vitality assay (MTT) in Caco-2 (left graph) and MCF-7 (right graph) cell lines treated 24, 48 and 72 h with different concentrations of PDS (0 – 100μM). b) Vitality assay (MTT) in Caco-2 (left graph) and MCF-7 (right graph) cell lines treated 24, 48 and 72 h with different concentrations of CX-5461 (0 – 20μM). c) IF staining of Caco-2 cells, treated 24, 48 and 72 h with different concentrations of PDS (0 – 50μM), and stained with BG4 antibody (green), and DAPI (Nucleus border is defined by white borders). Scale bar, 10 µm. Below, quantification of BG4 signal in the nucleus of the cells. d) IF staining of MCF-7 cells, treated 24 h with different concentrations of PDS (0 – 50μM), and stained with BG4 antibody (green), and DAPI (Nucleus border is defined by white borders). Scale bar, 10 µm. Below, quantification of BG4 signal in the nucleus of the cells. The graphs in c) and d) show mean fluorescence intensity (MFI) levels normalized over untreated cells of n=3 biological independent experiments. Horizontal line represents the mean value. Significance was determined using an ordinary one-way ANOVA multiple comparison. Asterisks indicate statistical significance; in detail, *P < 0.05, **P < 0.01, ***P < 0.001, ****P < 0.0001. [file 12915_2023_1805_MOESM3_ESM.pdf]

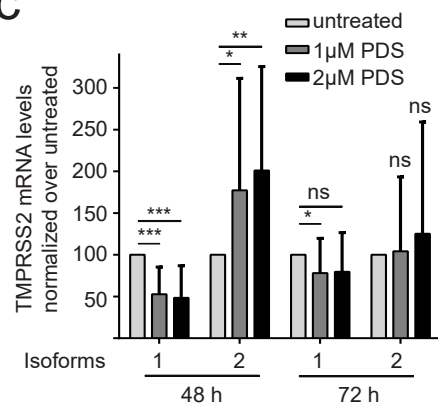

Supplement: Supplementary file 4 — Additional file 4. Treatments with CX-5461 induce G4s stabilization. a) IF staining of Caco-2 cells, treated 24, 48 and 72 h with different concentrations of CX-5461 (0 – 2μM), and stained with BG4 antibody (green), and DAPI (Nucleus border is defined by white borders). Scale bar, 10 µm. Below, quantification of BG4 signal in the nucleus of the cells. b) IF staining of MCF-7 cells, treated 24, 48 and 72 h with different concentrations of CX-5461 (0 – 2μM), and stained with BG4 antibody (green), and DAPI (Nucleus border is defined by white borders). Scale bar, 10 µm. Below, quantification of BG4 signal in the nucleus of the cells. The graphs in a) and b) show mean fluorescence intensity (MFI) levels normalized over untreated cells of n=3 biological independent experiments. Horizontal line represents the mean value. Significance was determined using an ordinary one-way ANOVA multiple comparison. Asterisks indicate statistical significance; in detail, *P < 0.05, **P < 0.01, ***P < 0.001, ****P < 0.0001. c) Expression levels of isoform 1 and 2 in MCF-7 cell line treated 48 and 72 h with 1 or 2μM PDS. mRNA levels in the graphs were normalized to the level of U6 snRNA and GAPDH. WT mRNA levels were scaled to 100%. Error bars represent SEM of at least n=3 biological independent experiments. [file 12915_2023_1805_MOESM4_ESM.pdf]

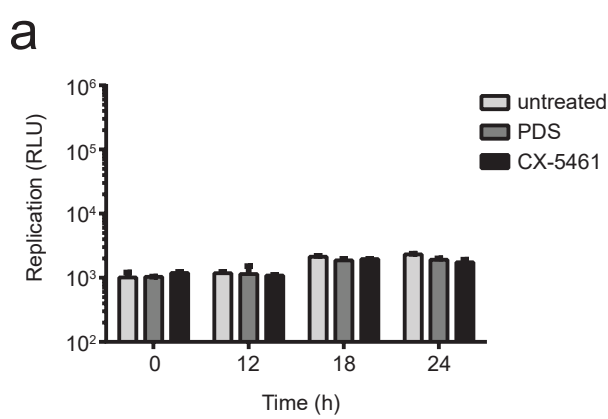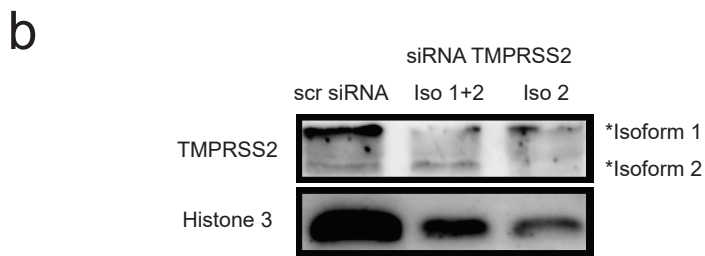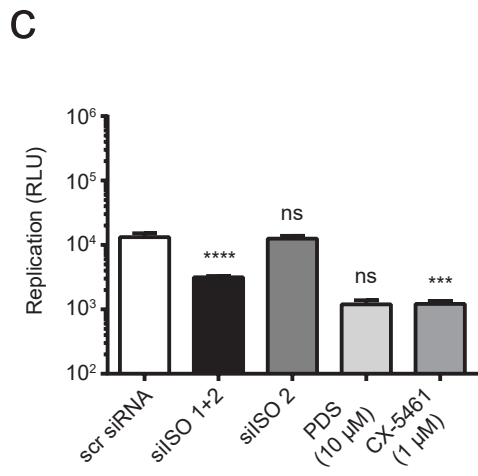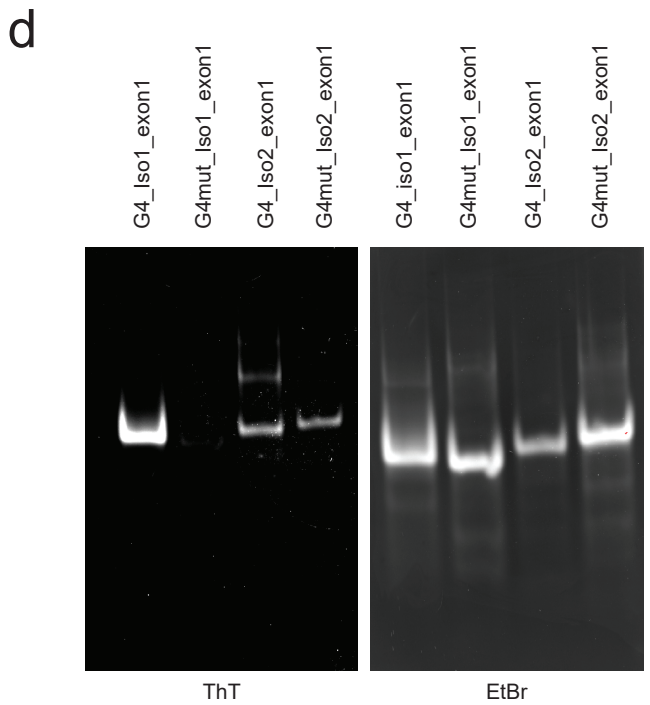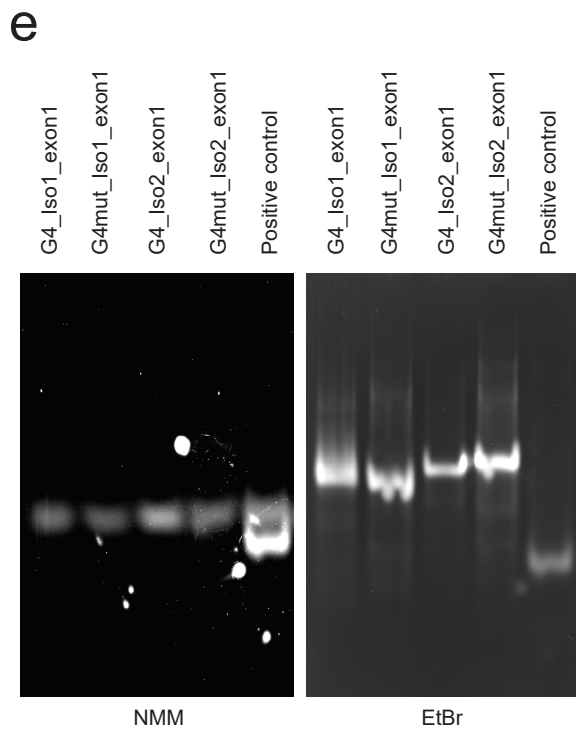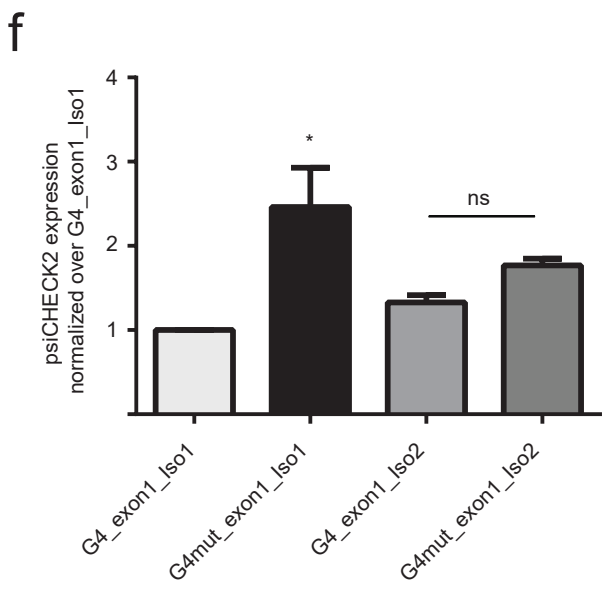

Supplement: Supplementary file 5 — Additional file 5. G4s modulate TMPRSS2 expression and SARS-CoV-2 replication. a) Time course experiments of MCF-7 cells pre- treated 24 h with 1μM CX-5461 or 10μM PDS and electroporated with the SARS-CoV-2 non-infectious replicon [52]. Luciferase plate was read at time points 0, 12, 18 and 24 h. b) Western blot analysis of protein extracts from Caco-2 cells transfected with siRNAs against TMPRSS2 isoform 1 and 2 and only isoform 2. Membrane was stained with anti-Tmprss2 and anti-Histone 3 antibodies. The original gel is reported in Additional file 9 c) SARS-CoV-2 replication in Calu-3 cells transfected with siRNAs against TMPRSS2 isoform 1 and 2, only isoform 2 and scramble siRNA or treated 24 h with PDS (10 μM), CX-5461 (1 μM), and Camostat Mesylate (25μM). Luciferase activity was measured 24 h after electroporation with the SARS-CoV2 non-infectious replicon [52]. d) ThT (left panel) and EtBr (right panel) staining of the oligonucleotide harboring G4s in the exon1 of the isoform 1 and 2 as well as oligonucleotide with mutations in order to disrupt the G4 motifs run in a 15% TBE native gels. e) NMM (left panel) and EtBr (right panel) staining of the oligonucleotide harboring G4s in the exon1 of the isoform 1 and 2 as well as oligonucleotide with mutations in order to disrupt the G4 motifs run in a 15% TBE native gels. f) HeLa cells transfected with psiCHECK™-2 vector containing DNA oligonucleotides harboring the predicted G4 motifs at the exon 1 of the isoform 1, the G-rich sequence in the isoform 2 as well as control mutated sequences cloned upstream of the reporter gene. Expression of psiCHECK™-2 was normalized to the G4_exon1_Iso1. [file 12915_2023_1805_MOESM5_ESM.pdf]

a

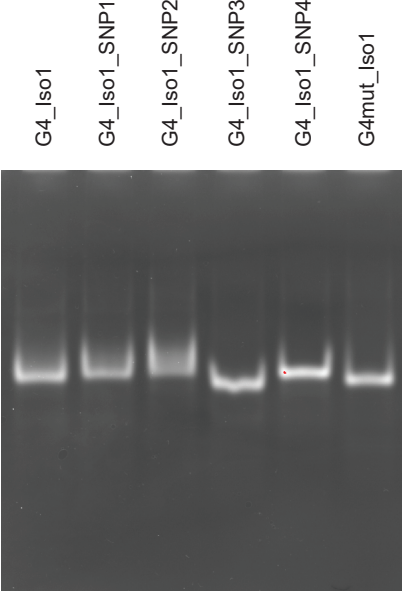

EtBr

b

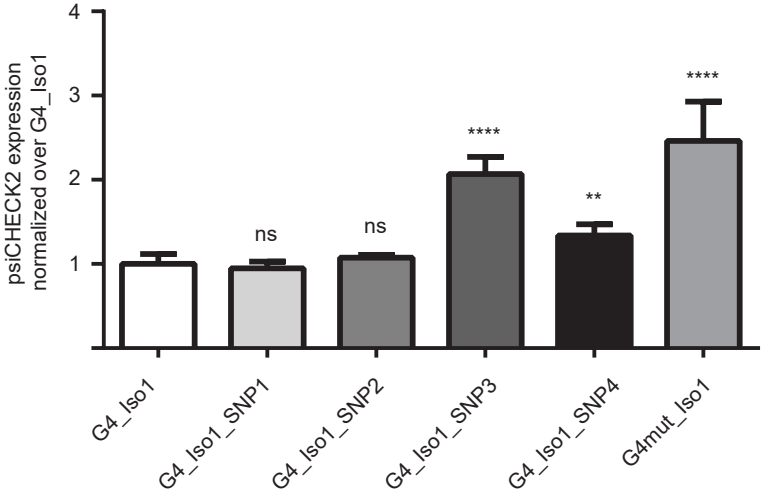

Supplement: Supplementary file 8 — Additional file 8. SNPs in the exon 1 (5´UTR) of TMPRSS2 isoform 1 influence protein expression in HeLa cells. a) EtBr staining of the oligonucleotide harboring the G4 in the exon1 of the isoform 1 as well as oligonucleotide contained four SNPs that partially disrupt the G4 motifs obtained by NCBI. b) HeLa cells transfected with psiCHECK™-2 containing the G4 at exon 1 of TMPRSS2 gene and the four SNPs that partially disrupt the G4 motifs obtained by NCBI, cloned upstream of the reporter gene. Expression of psiCHECK™-2 was normalized to the G4_ Iso1. [file 12915_2023_1805_MOESM8_ESM.pdf]

a

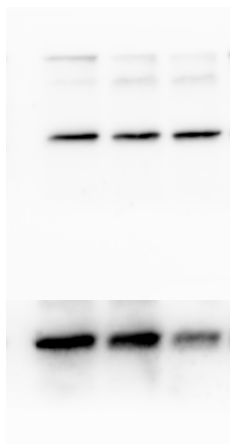

b

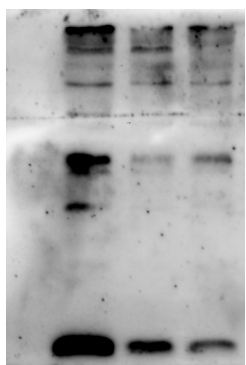

Supplement: Supplementary file 9 — Additional file 9. Original gels. a) original gels from Fig. 2e. b) original gel from Additional file 5. [file 12915_2023_1805_MOESM9_ESM.pdf]

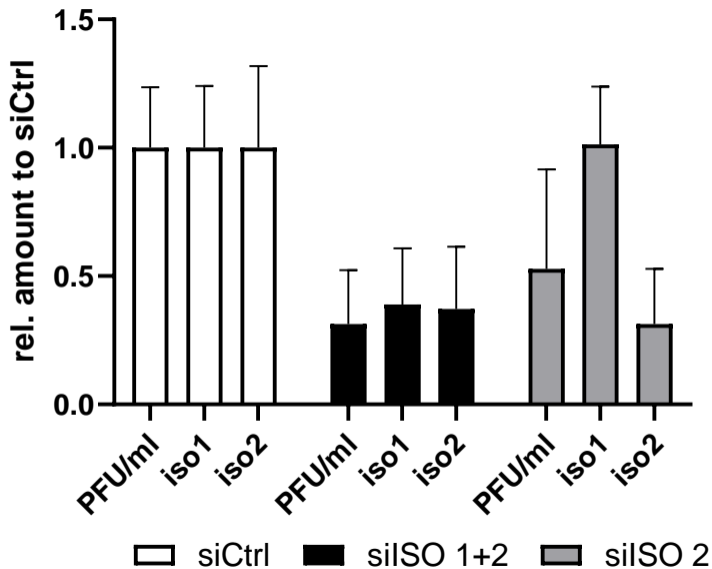

Supplement: Supplementary file 10 — Additional file 10. Infectious virus production and TMPRSS2 expression. Comparison of virus production (plaque-forming units (PFU)/ml) and knock-down efficacy of TMPRSS2 isoforms (iso1 and iso2) upon treatment with siRNA targeting both isoforms (siISO 1+2) or only isoform 2 (siISO 2). All values were normalized to siCtrl. [file 12915_2023_1805_MOESM10_ESM.pdf]
