# Supplementary material for: TMPRSS2 isoform 1 downregulation by G-quadruplex stabilization induces SARS-CoV-2 replication arrest
Source: BMC Biol. 2024 Jan 8;22:5. doi: 10.1186/s12915-023-01805-w (PMC10773119; doi:10.1186/s12915-023-01805-w)
Supplement: Supplementary file 6 — Additional file 6. DNA oligonucleotides harboring the G4 motifs in the exon 1 of the isoform 1 and in a G-rich region of isoform 2. as well as control sequences containing mutations within the PQS that are predicted, in silico, to prevent G4 formation. [file 12915_2023_1805_MOESM6_ESM.pdf]

	sequence
G4_5´-UTR_iso1	ccggctcggggtccgggctggggaggggaacctgggcgcctgggac
mutG4_5´-UTR_iso1	ccggctcgccgtccgcgctgccgagccgaacctgcgcgcctgcgac
G4_5´-UTR_iso2	caggaggcggaggcggaggcggagggcgaggggcggggagcgccgcctgg
mutG4_5´-UTR_iso1	caggagccgcagccgcagccggagcgcgagccgcgccgagcgccgcctcg

position
chr21: 41,508,011 - 41,508,056
chr21: 41,508,091 - 41,508,140
