# Supplementary material for: TMPRSS2 isoform 1 downregulation by G-quadruplex stabilization induces SARS-CoV-2 replication arrest
Source: BMC Biol. 2024 Jan 8;22:5. doi: 10.1186/s12915-023-01805-w (PMC10773119; doi:10.1186/s12915-023-01805-w)
Supplement: Supplementary file 7 — Additional file 7. DNA oligonucleotides harboring the predicted G4 motifs within the exon 1 of the isoform 1 as well as all 4 sequences containing one of the selected SNP. [file 12915_2023_1805_MOESM7_ESM.pdf]

	sequence
G4_5´-UTR_iso1	ccggctcggggtccgggctggggaggggaacctgggcgcctgggac
SNP1	ccggctcggggtccgagctggggaggggaacctgggcgcctgggac
SNP2	ccggctcggggtccgggctggggaggagaacctgggcgcctgggac
SNP3	ccggctcggggtccgggctggggaggggaacctggacgcctgggac
SNP4	ccggctcggggtccgggctggggaggggaacctgggcgcctgagac

position	NCBI reference
chr21: 41,508,011 - 41,508,056	
	rs1430156730
	rs565468881
	rs2091471351
	rs2091471316
